# In search for the sources of plastic marine litter that contaminates the Easter Island Ecoregion

**DOI:** 10.1038/s41598-019-56012-x

**Published:** 2019-12-23

**Authors:** Simon Jan van Gennip, Boris Dewitte, Véronique Garçon, Martin Thiel, Ekaterina Popova, Yann Drillet, Marcel Ramos, Beatriz Yannicelli, Luis Bravo, Nicolas Ory, Guillermo Luna-Jorquera, Carlos F. Gaymer

**Affiliations:** 1 Millennium Nucleus for Ecology and Sustainable Management of Oceanic Islands (ESMOI), Coquimbo, Chile; 2 Departamento de Biología Marina, Universidad Católica del Norte (UCN), Coquimbo, Chile; 3 Laboratoire d’Etudes en Géophysique et Océanographie Spatiales (LEGOS), Toulouse, France; 4 Mercator-Océan International (MOI), Ramonville, France; 5 Centro de Estudios Avanzados en Zonas Áridas (CEAZA), Coquimbo, Chile; 6 National Oceanography Centre (NOC), Southampton, UK; 7 Centro de Innovación Acuicola Aquapacífico, Coquimbo, Chile; 8 Cluster of Excellence “The Future Ocean”, GEOMAR Helmholtz Centre of Ocean Research Kiel, Marine Ecology Department, Düsternbrooker Weg 20, 24105 Kiel, Germany

**Keywords:** Environmental impact, Physical oceanography

## Abstract

Subtropical gyres are the oceanic regions where plastic litter accumulates over long timescales, exposing surrounding oceanic islands to plastic contamination, with potentially severe consequences on marine life. Islands’ exposure to such contaminants, littered over long distances in marine or terrestrial habitats, is due to the ocean currents that can transport plastic over long ranges. Here, this issue is addressed for the Easter Island ecoregion (EIE). High-resolution ocean circulation models are used with a Lagrangian particle-tracking tool to identify the connectivity patterns of the EIE with industrial fishing areas and coastline regions of the Pacific basin. Connectivity patterns for “virtual” particles either floating (such as buoyant macroplastics) or neutrally-buoyant (smaller microplastics) are investigated. We find that the South American shoreline between 20°S and 40°S, and the fishing zone within international waters off Peru (20°S, 80°W) are associated with the highest probability for debris to reach the EIE, with transit times under 2 years. These regions coincide with the most-densely populated coastal region of Chile and the most-intensely fished region in the South Pacific. The findings offer potential for mitigating plastic contamination reaching the EIE through better upstream waste management. Results also highlight the need for international action plans on this important issue.

## Introduction

Contamination of the oceans by plastic marine debris has become a widespread problem, recognized at the highest political level^1,2^, that threatens the natural balance of marine ecosystems^3–5^. Tackling marine litter pollution in specific regions, such as remote islands — where incoming plastics have travelled long distances — requires an understanding of the origins of these plastics so that effective waste management strategies can be put in place^6^.

However, our understanding of plastic transport and behavior in the ocean is not complete. Plastic marine debris (PMD) characteristics (shape, size, polymer density, etc…) play a key role in how PMD get transported by ocean currents. While surface 2D pathways for floating PMD have been largely documented they do not cover all potential pathways, as only a small fraction of annual input remains at the surface^7–10^. PMD size and buoyancy influence the level of exposure to wave and wind effects, but also how fast PMD resurfaces following transport at depth by vertical mixing induced by wind^11^. Other processes contribute to PMD not being solely constrained to the surface. UV degradation, biodegradation, ingestion by organisms or biofouling are processes that contribute to PMD fragmentation and alteration of its buoyancy^12^. Below a certain size (<200 μm), plastic debris are more evenly distributed in the mixed layer due to turbulence becoming dominant over buoyancy in setting the vertical distribution of plastic debris^13^. Further, micro-sized plastics are more prone than larger ones to be removed from the surface due to biofouling^14,15^. Beside inducing sinking, biofouling can also generate more complex oscillatory vertical movement as organisms growing make PMD heavier where conditions are favourable, i.e. at the surface, and lighter due to mortality where they are not, i.e. at depth^16^. Slightly-less-buoyant-than-seawater PMD are suspended at subsurface. These processes impact how PMD is advected by the ocean currents. Going beyond 2D surface pathways, recent studies have investigated other potential ocean-circulation pathways by examining 3D^6,17^ and subsurface 2D transport^17^. Developing an understanding of the origins of incoming PMD requires the inclusion of all transport pathways and potential polluting sources.

Remote islands can be highly contaminated by plastic debris waste produced elsewhere and carried across by the ocean currents. Connectivity with densely-populated upstream coastal zones, which are seen as important region of plastic input in the ocean, plays an important role in an island’s exposure to such pollution^6^. In addition, plastic debris may originate from marine-based activities that account for an estimated 20–30% of plastic-waste disposed in the oceans^7,18^ with commercial fishing being the dominant contributor, and spanning more than half of the global ocean^19^.

The case of Easter Island (also known as Rapa Nui) within the South East Pacific is of great concern as it hosts one of the most pristine marine ecosystems – owing to its isolation and nearby chain of sea mounts and islands – and as it suffers greatly from pollution of ocean-transported plastic marine debris. Easter Island and its Ecoregion (EIE), the area within 200 nautical miles of the Easter Island and the uninhabited Salas & Gomez Island, is situated to the west of a zone of convergence and accumulation for floating plastics^20^ where an average of 25,000 microplastics per km^2^ (sized 0.33 mm to 200 mm) populate the surface^21,22^. Within the waters of the EIE, high densities of floating macroplastics have been reported^23^. Many studies show markedly higher levels of plastic ingestion for fish species (as high as 80% of sampled fish) near Easter Island due to its environment of high microplastic concentration and low food availability^24,25^, relative to other regions of the South Pacific. Birds, marine mammals and sea turtles are also frequently affected by plastic ingestion^26,27^. Beach surveys report extraordinarily high plastic debris density^28^. Such densities are equivalent to or higher than densities observed on another island of the South Pacific, the inhabited Henderson Island, where extensive surveys were carried out^29^. It is difficult to determine the potential continental origin of plastic litter because the vast majority of identifiable objects found on the island’s beaches (Easter Island and Salas y Gómez) are from fishing activity^26–28,30^ (e.g. nets, buoys, crates…) with the rest consisting of fragmented debris.

The majority of plastic debris with terrestrial origin found in the EIE is likely limited to countries surrounding the South-Pacific basin^8^. Although inter-ocean exchanges exist^31^ for instance influx from the Indian Ocean^32^, their associated timescales may be considered too long (tens to a hundred of years) relative to the plastic debris residence time in the surface layer^10^ to introduce large amounts of plastics from other basins. Marine-based sources of the South-Pacific, identified as intense commercial fishing regions, are located within productive upwelling regions such as the Peru upwelling region and the tuna-abundant regions of the equatorial and subtropical Pacific (Fig. S1).

Here the framework of ref. ^6^ for identifying exposure to upstream potential polluters is applied to the remote ecoregion of Easter Island with connectivity to both land- and marine-based sources examined. We use a Lagrangian approach coupled to an eddy-resolving ocean general circulation model (OGCM) to populate and backtrack the trajectories of “virtual” passive particles reaching the EIE. For a comprehensive approach, we investigate both the transport pathways induced by surface oceanic currents and full three-dimensional ones. We evaluate the results’ sensitivity to long-term variability of the Pacific Ocean currents by investigating other periods of OGCM simulations. The influence of mesoscale activity using OGCM with different resolution (eddy permitting and resolving) and forcing is also scrutinized. We use population density and fishing activity datasets to diagnose exposure to both land- and marine-based sources of plastic waste and propose target regions along the South American west coast for putting in place effective waste management and reduce the influx of plastic to the EIE.

It has been suggested that contamination by litter is likely inversely proportional to the distance to the nearest gyre centre that corresponds to the region with highest litter density^26,33^, implying that these “garbage patches” are the main source of contamination. However, this approximation does not consider potentially important pathways of marine litter operating at shorter timescales (seasonal, or multiyears) and assumes that plastic litter accumulates within the garbage patch prior to contaminating neighbouring regions, which may not be the case when island locations intersect the main routes of plastic litter. For example, plastic litter deposited by industrial fishing boats may strand on island shores before having reached the centre of the gyre accumulation zone. The focus of this paper is on those “fast” pathways of plastic litter. We consider timescales consistent with basin-scale connectivity (<10 years) to diagnose the short time (relative to the lifetime of plastics) pathways of plastic transport to the EIE.

## Materials and Methods

### High-resolution global circulation models

In this study, the Lagrangian experiments use velocity 5-daily field outputs from global ocean circulation simulations issued from the Nucleus for European Modelling of the Ocean (NEMO) framework^34^. We use the high-resolution hindcast free-run simulation, run at the National Oceanographic Centre, Southampton (NOC), which covers the period from 1958 to 2015. The model has a horizontal resolution of a 1/12 ° and has 75 vertical layers varying in thickness progressively increasing from 1 m at the surface (with 22 levels in the top 100 m) to 250 m at 5500 m depth, with partial steps used to represent the depth of each bottom cell using as reference the bathymetric database ETOPO2^35^ in the open ocean and GEBCO for the continental shelves^36^. The model is forced with the atmospheric Drakkar Forcing Dataset version 5.2^37^. Further details about this run are available in ref. ^38^. We refer to this simulation as NOCS12. In this study, we used the velocity field for the period 2006–2015 for the Lagrangian experiment. The experiment was repeated for the period 1968–1977 and 1990–1999 to quantify the sensitivity of results to the decadal variability in ocean circulation. In particular, the Interdecadal Pacific Oscillation (IPO) has a strong loading on sea surface temperature in the South Eastern Pacific^39,40^ and is associated to a low-frequency modulation of the gyre circulation^41,42^, potentially impacting the characteristics of the main pathways of plastic debris that are documented here.

While NOCS12 uses a realistic atmospheric forcing, it still presents biases associated with imperfect model physics and to biases in the forcing itself, in particular in the coastal wind profiles^43^. In addition, since it does not use assimilation of data, the turbulent flow (mesoscale eddies) is not constrained so that only the statistical properties of the turbulence can be compared to observations. In general, assimilated products provide more realistic velocity fields at mesoscale, although this can be sensitive to resolution and assimilation schemes. Since the mean circulation is weak in our region of interest (compared to western boundary systems and equatorial regions), the mesoscale flow is relatively energetic, with a main source originating from the instability of the coastal currents along the coast of South America^44–46^. The connectivity patterns inferred from our Lagrangian experiments can be therefore sensitive to the characteristics of the mesoscale flow. In order to evaluate the sensitivity of the circulation pathways to ocean mesoscale flow produced by various modelling approaches, two oceanic reanalyses were used for the Lagrangian experiment. These reanalyses, which differ from free-run models in that they assimilate *in situ* and satellite-derived observations of the ocean, are based on the same OGCM as NOCS12 (NEMO), and are developed by Mercator-Océan in the framework of Copernicus Marine Environment Monitoring Service (CMEMS). The first, GLORYS2V4, has a horizontal resolution of 1/4° with 3-daily mean outputs; and the second, GLORYS12V1, one of 1/12° with daily outputs, and both cover the period 1993–2015. The reader is referred to ref. ^47^ and ref. ^48^ for more details about the model configurations and validation. Here, since we are interested in evaluating how these products simulate the mesoscale activity in our region of interest, we compare the mean eddy kinetic energy (EKE) at the surface (a measure of the magnitude of mesoscale activity) for all model simulations with EKE estimates based on satellite altimetry over the period 1993–2014 (Fig. S2). Significant dispersion exists amongst the simulations in terms of the EKE level. While NOCS12 simulates amplitude for mean EKE that is comparable to observations off the coast of Peru, it tends to overestimate it off northern Chile. On the other hand the Mercator-Océan products tend to underestimate the mean EKE almost everywhere, which is inversely proportional to the resolution of the model as expected. However the pattern of mean EKE is more realistic in the Mercator-Océan products owing to the assimilation of the altimetry data. The spatial correlation between Mercator-Océan products and observations reaches 0.92 (0.93) for GLORYS2V4 (GLORYS12V1) in the latitude range between 40°S and 20°S, which is larger than the one for NOCS12 (c = 0.81). The root mean square difference between observations and models reaches 96.36 cm/s^2^ (NOCS12), 87.25 cm/s^2^ (GLORYS2V4) and 65.99 cm/s^2^ (GLORYS12V1) over the same domain. The three simulations, although based on the same oceanic model, provide distinctly different patterns of the mesoscale flow, which can be used to evaluate the sensitivity of our results to the representation of the eddy activity.

### Lagrangian particle tracking: experimental setup

To model the dispersal of plastic marine debris we use a Lagrangian modelling approach with the particle-tracking tool ARIANE^49^. For this method, “virtual particles” are seeded at a given time and place in the model flow field and their trajectories derived over time by updating the particles’ positions after each time step of the model output according to the mean velocities associated to the grid cells wherein particles lie.

This method presents the advantage to run Lagrangian experiments offline therefore enabling the comparison of a Lagrangian experiment carried out using model runs with different configurations. Also, particles for a given site can be seeded at multiple times across the modelled period, and at multiple slightly offset neighbouring locations, and generate a set of trajectories containing a wide range of temporal and spatial variability. The superimposing of all trajectories can reveal how connected a site is with its surroundings (here for instance Easter Island with plastic waste generating hotspots) and how well defined these connection pathways are.

**Figure 1:**
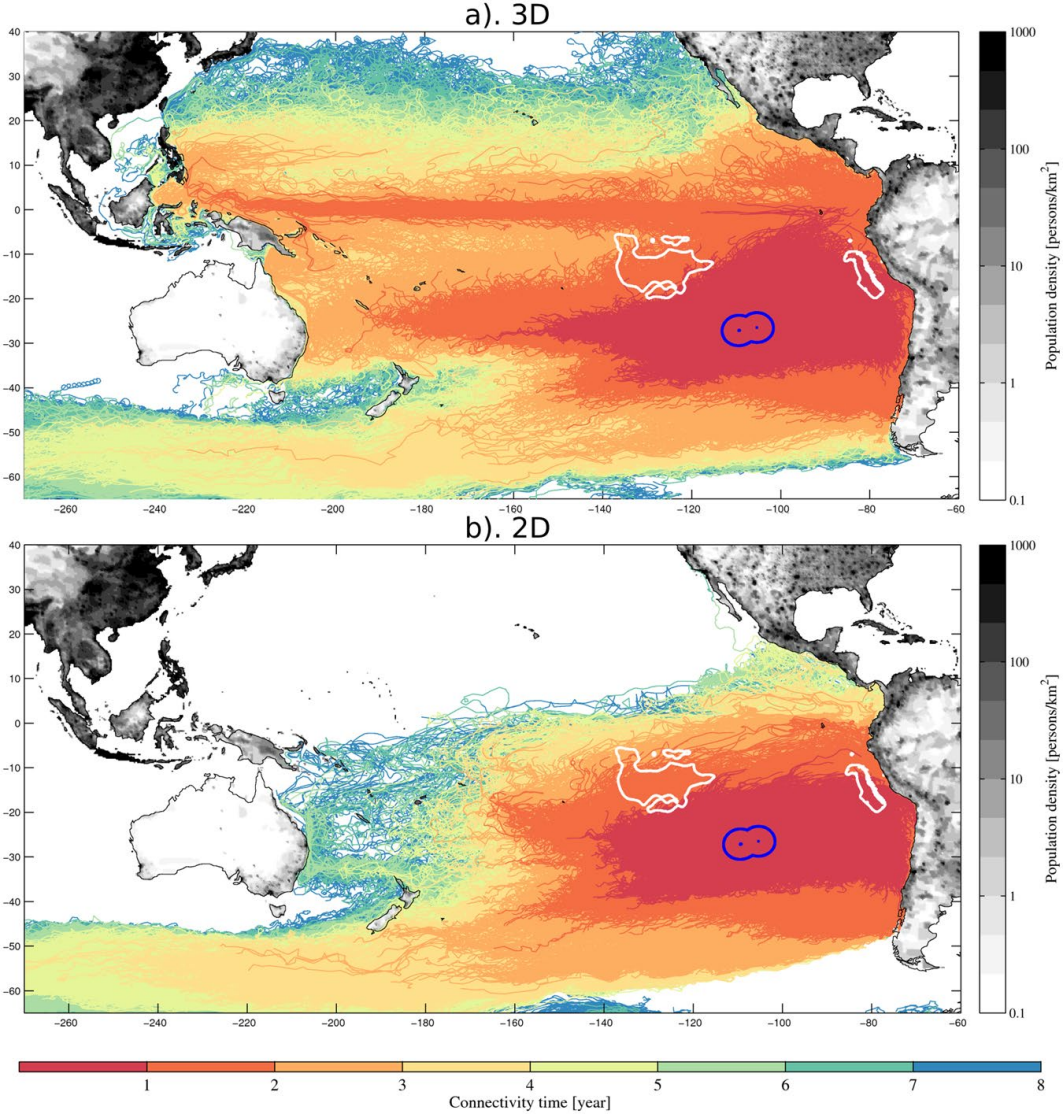
Ensemble of trajectories released within Easter Island Exclusive Economic Zone (EEZ, blue contour) across the decade 2006–15 and advected backwards in time for 8 years in the 3D (**a**) and 2D flow (**b**). Trajectories are multicolored with each color representing the portion travelled over the course of the associated year before arriving to destination within the EEZ, e.g., in red the segment of trajectories covered within the first year, orange in the 2nd, etc… Note that segments for each year are superimposed and therefore show the largest extent of the dispersal for each year. White contours represent the two intensive commercial fishing zones — Central Pacific and offshore from Peru — investigated in this study. On land, darkest grey colors represent regions of high population density.

To examine the origin of water parcels reaching the EIE – that potentially carry plastic debris – and therefore the EIE’s exposure to plastic waste hotspots, particles are released in the modelled oceanic flow within EIE and advected in backward mode such that their positions are backtracked in time. Effectively, the location where we seed particles corresponds to the particles’ destination, and where they get advected to, corresponds to their origin. The set-up is inspired from the study of ref. ^6^. We use the island’s Exclusive Economic Zone (EEZ) – whose limits are also those of the Ecoregion – as boundaries for the releases (Fig. 1) and use a period of 8 years for the advection by the flow of the particles. Particles are released monthly for the entire decade 2006–2015. This range of release dates results in particles being transported for different periods: for instance a particle released on 1st of January 2006 is tracked back to 1st of January 1998; one released on the 1st December 2015, to the 1st December 2007.

Two types of Lagrangian experiments are carried out. The first one uses the three-dimensional current flow to advect particles, and therefore enables them to travel through the water column. It is made to be most representative of neutrally buoyant particle debris, most notably microplastics. The second one uses only the sea surface currents and as such constrains the particles to the surface. It is made to represent buoyant, predominantly macro-sized, plastic debris. For the 3D experiment, particles were placed at ~50 km interval horizontally within the EIE and at depth of 0 m, 20 m, 40 m and 60 m depth – equally spaced across the mixed layer depth of yearly mean value 60–70 m in the EIE – resulting in 1712 particles (4 layers, each containing 428 particles). With monthly releases across the period 2006–2015, a total of 205,440 particle trajectories were generated (Fig. 1). For the 2D experiment, only releases within the first vertical grid layer (0.5 m) were made. Spacing between release sites was reduced to 20 km to enable more releases, generating 2,669 locations wherefrom a total of 320,080 particles were deployed. Independent of the frequency of the output of the model studied, trajectories of particles are recorded at 5-daily frequency. The timescales involved with plastic transport (order of years) do not require a finer time resolution.

### Connectivity of easter island ecoregion with plastic waste generation regions

#### Identifying plastic waste generation hotspots

To assess the exposure to plastic waste pollution transported by the ocean circulation to Easter Island, we identified land and marine hotspots of plastic waste generation using as index coastal population density and industrial fishing pressure (Fig. S1). Although Easter Island is located near a plastic accumulation zone, and that a fraction of the plastic polluting EIE waters could come from this accumulation zone, we do not consider it as a source of plastic waste in this study. It consists of long-term plastic accumulation and represents an indirect source. Here the focus is set on identifying well-defined “fast” pathways of plastic debris to the EIE within the South Pacific basin and, thereby, direct plastic sources only.

##### Population density

Population density has commonly been used as an index for determining plastic input in the ocean^8,9,50^ with densely populated coastal areas considered as plastic waste generation hotspots. Population density used in this study are the 1/4° resolution Gridded Population of the World (Version 3, GPWv3) projections for 2015^51^ produced by the Center for International Earth Science Information Network (CIESIN) and the Centro Internacional de Agricultura Tropical (CIAT). Coastal regions of the Pacific basin present highly heterogeneous population density (Fig. 1).

##### Global fishing pressure

Significant proportions, estimated to be around 20% by number and/or 70% by weight^20^, of plastic waste entering the ocean come from marine-based human activities and notably fisheries^2^. The Global Fishing Watch database^19,52^ was used as index for determining marine plastic generating hotspots, with the underlying assumption that the quantity of waste originating from a marine location is function of the time spent fishing in this area. The database quantifies the total fishing time per day per 1/100° grid cell for the period 2012–2016. We used the cumulative fishing time for each cell across the period and defined zones of intense fishing as the regions where fishing hours exceed 50 hours per grid cell over the 2012–2016 period (Fig. 1, S1).

#### Measuring connectivity

##### Connectivity with land-based plastic sources

The connectivity of EIE with other land regions of the South Pacific basin was determined by investigating trajectories of particles (deployed within the EIE and being advected backward in time) that travelled within 50 km of continental coastlines or islands. A 50 km distance from the coast was chosen because it is within the average width of global coastal margin^53^ and because velocities within that band are poorly resolved as the models are too coarse for representing well the shelf dynamics. Indeed, the 1/12° model grid only has 3–4 gridcells within that band (and just 1–2 gridcells for the 1/4° GLORYS2V4 model.

For each of these trajectories, the position where the particle first entered and the time taken was recorded. Results of these positions for all trajectories penetrating coastal areas were then grouped into 5° by 5° grid cells. The size of the box was chosen to be large enough to have trajectories travelling through them, but also sufficiently small to capture regional variability. The proportion of connecting particles out of the total deployed and the distribution of particles travel time was calculated to assess the strength and the timescale of the EIE’s connectivity with each coastal zone. This method presents the advantage that the sum of the proportions from each coastal zone equals 100% of the particles connecting with land, and permits to get a first order idea of the different origins of particles.

##### Connectivity with marine-based plastic hotspots

Two locations of intense fishing are found in the South Pacific near EIE: one just outside Peru’s EEZ, to its north-east, named Fishing Zone Peru (FZ-P), and one to its north-west in the middle of the Pacific, named Fishing Zone Central Pacific (FZ-CP) (see white contours in Fig. 1). To delimit these two regions we selected the 1/10th degree grid cells within which the cumulated fishing activity was larger than 50 hours for the period of 2012–2016 of the database. FZ-CP is 1.91 million km^2^ and located between 1750–4000 km from the EIE centre, a total of 1,101,396 hours of industrial fishing is recorded equivalent to 57.8 hours per 100 km^2^. FZ-P is in contrast smaller with 0.44 million km^2^ and further away from EIE (2800–3300 km) with a total of 366,339 hours of fishing recorded equivalent to 83.2 hours per 100 km^2^.

We identified the trajectories that entered each fishing zone, recorded the position on the boundary where it first entered and the time it took to get advected to reach the EIE. Connectivity with fishing zones were studied separately, therefore particles that pass through both fishing zone are included in the statistics for each fishing zone.

### Ethics statement

The paper does not include any sampling and laboratory analysis of marine organisms. Therefore, no ethics approval was required.

## Results

### Main features of the upstream circulation connectivity of easter island

Although EIE is a remote region, our results show that it is strongly connected to distant sources of plastic litter due to the ocean circulation connectivity. These include coastal regions of high population density such as continental Chile and regions of intense industrial fishing. Their connectivity with the EIE depends on the ocean circulation (Fig. 1).

In the experiment using the 3D flow, EIE is connected to the entire basin under the 8-year timescale (Fig. 1a). Particles are carried by ocean currents to the EIE from the northeast and connect the South American coast at around 10°S–35°S. Further upstream, particles arrive from the north or from the south. From the south via the northward flowing Humboldt Current with particles originating from the south east of the subtropical gyre or the Antarctic Circumpolar Current; from the north through the Peru-Chile Counter Current and Peru-Chile Under Current closer to the coast in the subsurface – both fed by the eastward-flowing Equatorial Undercurrent. Particles reaching the EIE from the (south) western side converge to the ecoregion through the slower eastward current of the gyre. In contrast, trajectories from the 2D experiment do not connect to the entire basin with most particles coming in from the south-eastern part (Fig. 1b). Results of the EIE connectivity to distant source of pollution are presented in the context of particle size, with the 2D experiment associated to floating macroplastics and 3D experiment to neutrally buoyant microplastics.

### Connectivity with land sources

**Figure 2:**
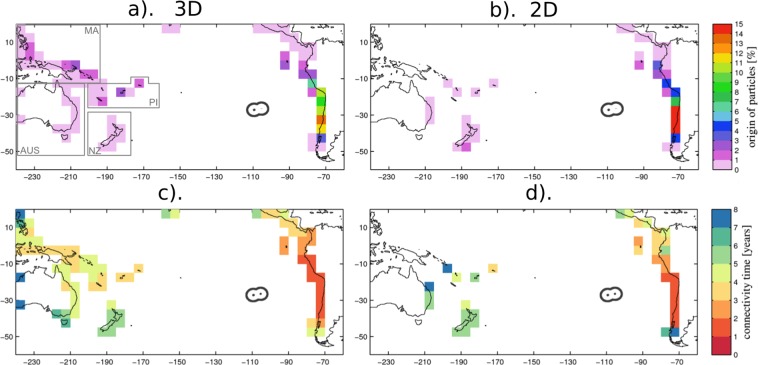
Strength of connectivity (**a**,**b**) of Easter Island Ecoregion with coastal zones of the Pacific basin — expressed as the percentage of particles originating from one given grid cell out of all particles originating from coastal zones— and connectivity time (**c**,**d**). Note that particles are deployed within Easter Island EEZ (black contour) and backtracked in time in the 3D (left) and 2D (right) flow. Only the first coastal zone that a particle connects to (i.e. the last one before reaching Easter Island) is considered in this method. Connectivity time is obtained by taking the 10th percentile from the distribution of the travel time taken by all particles connecting the given coastal zone and Easter Island. Areas squared in a) correspond to regions for which data is grouped together for inter-regional comparison in the analysis (Table 1), they include the Malay Archipelago (MA), Pacific Island (PI), Australia (AUS) and New Zealand (NZ).

In the 3D experiment, only 45% of particles can be traced to the coastal zones within the 8 years of the experiment (Fig. 2a,c). These are referred to as ‘land’ particles hereafter. The other 55% of particles have not been within 50 km of coastlines for the time period of the experiment (8 years) and have only travelled in open ocean waters. Trajectories of particles deployed around the EIE connect to all the coastal regions (here represented by 5 ° by 5 ° grid cells) of the South Pacific but the strength and time – measured by the proportion of land particles and 10^th^ percentile of the time distribution– of each regions’ connectivity with the EIE varies significantly. A marked difference exists between east-west origins of particles within the South Pacific. The east Pacific coast is source of 77.1% of particles originating from land, whereas only 22.8% are coming from the west (with Hawaii source of the other 0.1%). This is not surprising given the characteristics of the mean surface circulation and the shorter distance of the EIE from the South American coast. Connectivity times from the eastern side of the basin to the EIE are much shorter with timescales of 1.8 to 3 years for the east Pacific coast, as opposed to 4.2 to 6 years for the west.

At the regional scale, most of the 5° by 5° coastal regions of South America are sources of at least 3% of connecting land particles. These are concentrated between 5°S and 45°S, with a peak of 13% for 30°S–35°S. The band within 20°S–40°S is source of 47.5% of the total particles being traced back to land. In addition, this region also displays the fastest connectivity with the EIE, with travel times of 1.7 to 1.9 years (Fig. 2c). The Chilean coast is source of 49.8% of particles taking 1.8 years (Table 1). Further north connectivity progressively weakens in intensity and takes a longer period of time, e.g. Peru (20.6% and 1.8 years), Ecuador (5.1% and 2.2 years) and Colombia (0.5% and 2.4 years). Connectivity patterns and timescales show little year-to-year variability (Fig. S9).

**Table 1:** Summary for the statistics of the origins of particles deployed around Easter Island and advected backwards in time (as displayed in Fig. 2) for different model runs (NOCS12, GLORYS12, GLORYS2V4), advection modes (2D, 3D) and time periods.

Model	NOCS12		NOCS12		NOC12		NOCS12		GLORYS12		GLORYS2V4	
Experiment (mode, period)	3D (2006–15)		3D (1990–99)		3D (1968–77)		2D (2006–15)		2D (2006–15)		2D (2006–15)	
Proportion of released particles originating from land	45%		44%		48%		52%		68%		67%	
	%	Time (yrs)	%	Time (yrs)	%	Time (yrs)	%	Time (yrs)				
**West Pacific**	**22.8**		**18.3**		**13.3**		**2.5**		**1.3**		**0.7**	
Malay Archipelago	12.5	4.0	9.0	4.5	7.2	4.2	<0.001	7.9	<0.01	4.5	<0.001	6.7
Pacific Islands	8.0	3.7	6.8	4.2	5.0	4.2	<0.05	4.0	<0.1	4.5	<0.05	4.6
Australia	1.7	4.1	1.9	4.7	0.9	4.6	<0.05	5.4	<0.01	5.7	<0.05	5.7
NZ	0.6	5.5	0.6	5.2	0.3	5.2	2.5	5.2	1.2	5.1	0.7	4.6
**East Pacific**	**77.1**		**81.7**		**86.7**		**97.5**		**98.7**		**99.3**	
Central America	1.1	3.2	0.7	3.7	0.5	3.4	<0.1	3.8	1.4	3.0	0.2	3.5
Colombia	0.5	2.4	0.3	2.8	0.3	2.5	<0.1	3.5	0.4	2.9	<0.1	3.7
Ecuador	5.1	2.2	4.4	2.6	3.9	2.5	2.5	3.2	2.0	2.6	0.9	3.2
Peru	20.6	1.8	20.9	1.9	19.6	2.0	14.6	2.0	21.2	1.8	8.3	2.0
Chile	49.8	1.8	55.3	1.9	62.3	2.0	80.3	1.6	73.7	1.6	90.0	1.4
**Fishing Zone**												
Central Pacific	12.2	2.5	—	—	—	—	6.3	1.9	—	—	—	—
Peru	18.5	1.4	—	—	—	—	15.5	1.2	—	—	—	—

Terrestrial and marine sources are displayed. Terrestrial sources are organized in West and East Pacific coast, marine ones consist of Fishing Zone in the Central Pacific and off the Peru EEZ (see Fig. 1 in white contours). For the East Pacific coast results are detailed per coastal country; and for the West Pacific coast, per region (as delimited by the squared boxes in Fig. 2). Strength of connectivity is expressed as a percentage of total number particles originating from land for terrestrial sources, and as percentage of total particle released for fishing zones. Timescales correspond to the 10^th^ percentile of the time distribution obtained from trajectories’ time to connect EIE with the given region. Note in row 1 the proportion of the total number of released particles that originate from a coastal region for each experiment.

The western part of the South Pacific basin is source of the other 22.8% of land particles. Connections are the strongest with the Malay Archipelago region (see square in Fig. 2a) wherefrom 12.5% of land particles originate. The dominant pathway to the EIE occurs through the Equatorial Under-Current (EUC) and takes 4.0 years. Other sources in the west include the Pacific islands (PI), with 8.0% of land particles and connectivity times of 3.7 years. Australia and New Zealand are origin of 1.7 and 0.6% of land particles taking 4.1 to to 5.5 years connectivity time, respectively.

In the surface 2D experiment, more particles deployed within the EIE originate from land (52%), with a smaller portion (48%) never coming within 50 km from the continent or islands; the majority of the latter ones (never approaching coastal zones) originating from the southern Ocean (Fig. 1b). Of the particles contacting land, 97.5% come from the east Pacific coast and only 2.5% from the west (Fig. 2b,c and Table 1). 82.3% of particles originate from the South American coast between 15°S and 45°S, and 65.3% within 25°S–40°S. In contrast with the 3D experiment, a much smaller fraction of particles (<0.1%) originates from the Malay archipelago (MA) and Pacific Islands (PI). Focusing on the eastern side, we find stronger and shorter timescales of connectivity in the 2D experiment with the Chilean coast (80.3% and 1.6 years) and weaker and longer connectivity for countries further north e.g. for Peru (14.6% and 2.0 years) and Ecuador (2.5% and 3.2 years) (Table 1).

In summary, we find that the region along the coast of Peru-Chile within the latitude ranges of 15°S and 40°S is potentially a dominant source region of plastic contamination for the EIE independently of whether the surface or the 3D circulation is considered in estimating the connectivity pattern.

### Connectivity with intense fishing area sources

We now consider the two zones of intense industrial fishing in the neighbourhood of the EIE (Fig. 1): one located to the north-west of the island, and here called Central Pacific Fishing Zone (FZ-CP); and another, off the coast of Peru, denoted Peru Fishing Zone (FZ-P). The EIE does not show the same connectivity with these two intense fishing zones (Fig. S3).

**Figure 3:**
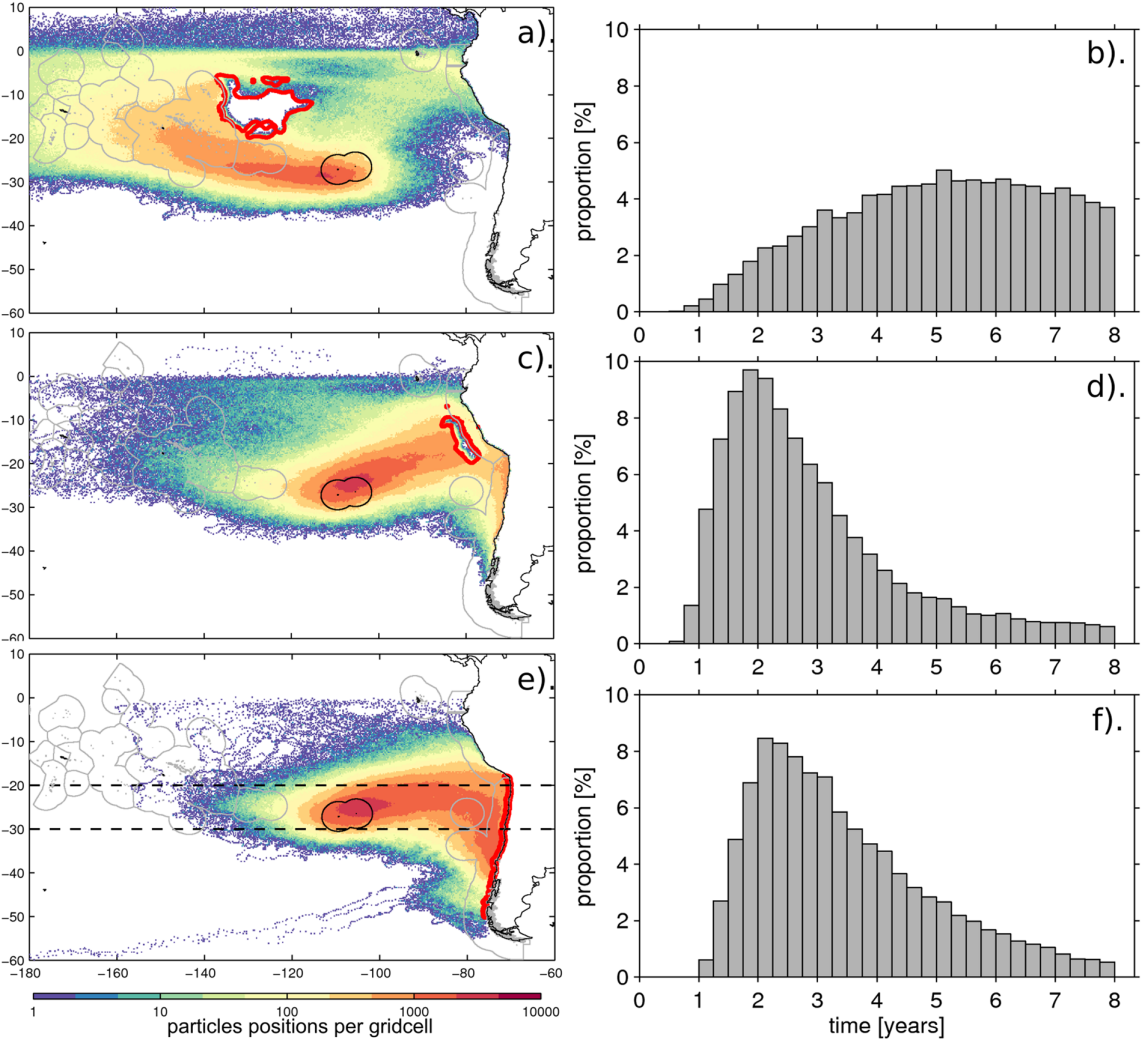
Connectivity footprint of Easter Island Ecoregion (EIE, solid black contour) and associated distribution of connectivity time with 3 potential plastic waste generating source regions (solid red lines), in the case of the 3D backward tracking experiment: the intense commercial Fishing Zones in Central Pacific FZ-CP (**a**,**b**), the one off the Peru Economic Exclusive Zone (**c**,**d**), and the Chilean coast (**e**,**f**). For each footprint (**a**,**c**,**e**), the 5-daily positions of particles connecting to the source region are binned into 0.25 ° by 0.25 ° grid cells. Individual trajectories are illustrated in Fig. S3. The distribution of time taken by particles to connect the source region to EIE (right) is normalised by the total number of connecting particles. Dashed lines in (**e**) are used in Fig. 5 to show a zonal section of the particles distribution with depth.

In the case of the 3D microplastic experiment, 18.5% of the deployed particles passed through FZ-P and 12.2% through FZ-CP. Many of the particles’ trajectories from FZ-P connect directly to the EIE (Fig. 3c,d), reaching this area faster than for FZ-CP for which particles get advected preferentially within the South Pacific and thus take more time (Fig. 3a,b). Time distribution of connecting particles for each fishing zone shows the faster connectivity for FZ-P (Fig. 3b,d,f). The time distribution for particles connecting FZ-P is skewed towards short timescales with 50% of particles connecting in less than 2.5 years. The fastest 10% – used here to define the connectivity timescale –takes less than 1.4 years. In contrast, the time distribution for particles connecting FZ-CP is more uniform and appears truncated at the end of the 8-year experiment, suggesting the length of the experiment is too short to obtain the full distribution. Statistics for FZ-CP are not fully representative, as they do not capture the complete distribution. They show that the connectivity is slower and that particles reach EIE by multiple and less-defined routes, some including a recirculation through the EUC (Fig. S3b). The fastest 50% of particles connecting the FZ-CP to the EIE take up to 5.2 years, with the fastest 10% less than 2.5 years.

Connectivity footprints for each fishing zone with the EIE are derived using connecting particles’ 5-daily positions and reveal the grid cells of the model that are most travelled through by these particles. These show a well-defined pathway between the FZ-P and the EIE (Fig. 3c) with a broad continuous network of grid cells containing more than 750 particles’ positions linking both sites in a straight line. In contrast, pathways between the EIE and the FZ-CP are not as well defined (Fig. 3a). Particles reach the EIE using various pathways, either direct ones which are fastest or more indirect ones: clockwise via the Equatorial Under Current reaching the Peru coast and then westward to Easter Island, or counter-clockwise via the slower subtropical gyre circulation (Fig. S3b).

With fisheries producing mostly floating macroplastic waste, the surface 2D experiment is particularly relevant for measuring connectivity of Easter Island. Connectivity patterns are comparable to the 3D experiment (Fig. S4) although they are weaker with only 6.3% and 15.5% of particles connecting with the FZ-CP and FZ-P, respectively. Overall connectivity timescales are shorter due to particles being constrained to the surface layer where currents are faster. For FZ-CP, a difference in connectivity exists with a better-defined pathway to the EIE for the surface than in the 3D case. This is also apparent in the time distribution which is unimodal and well captured by the 8-year experiment, with 50% of particles reaching EIE under 3.3 years and fastest 10% in less than 1.9 years. For FZ-P, connectivity patterns are very similar to 3D but with faster connectivity times: 50% of particles connect in less than 2.1 years, and fastest 10% in less than 1.2 years.

This study assesses EIE connectivity with coastal regions of the Pacific basin and suggests that the South American coast, and in particular the Chilean coast, is a potentially important source of plastic contamination for the EIE.

### Sensitivity of results to mesoscale and decadal variabilities

**Figure 4:**
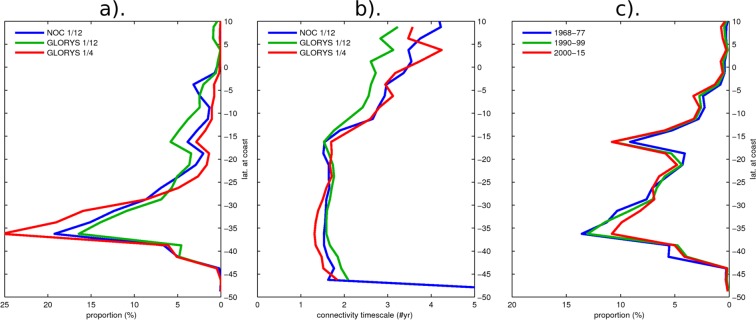
Focus on particles connecting Easter Island Ecoregion to the South American coast. Panel (a,b) Sensitivity of connectivity strength and associated timescale to mesoscale turbulence evaluated by carrying out the Lagrangian 2D experiment in velocity fields of model simulations with different configurations: NOC12 (blue), GLORYS12 (green) and GLORYS2V4 (red). Panel (c) sensitivity of connectivity strength (in 3D flow) to decadal variability with different Inter Decadal Oscillations periods investigated.

The mesoscale circulation in the form of eddies and fronts influences the horizontal oceanic transport and may impact the connectivity of the EIE with the South American Continent. As an attempt to assess the sensitivity of our results to the representation of the mesoscale activity, the results of the connectivity experiment investigating 2D (floating macroplastic) transport pathways are compared with identical experiments carried out with outputs of ocean models differing in their simulated patterns of mean EKE (Fig. S2; see section 2 for details in the models’ configuration). Results are presented in Table 1 and Fig. S8 with focus on the South American coast in Fig. 4a,b. Both assimilated runs show increased particles originating from land with 68% and 67% respectively for GLORYS12 and GLORYS2V4., Peru and Chile combined are the origin of 94.9% and 98.3% of particles originating from the coast of South America for GLORYS12 and GLORYS2V4, respectively, relative to 94.9% for NOCS12. Connectivity times for this region are similar (1.6 to 2.0 years). The model with the lower resolution (GLORYS2V4) yields a somewhat stronger connectivity, consistent with the notion that mesoscale activity would tend to have a diffusive effect on the connectivity. It is noteworthy that, while at eddy-resolving resolution, our model setup does not resolve realistically the coastal dynamics. It does not include a number of processes (e.g., vertical mixing by boundary-layer turbulence, internal tides dissipation, inertia-gravity wave, bores…) that could be influential for the transport of plastic litter from coast to the open ocean. That is why our method for calculating the number of particles connecting to a source region focused, for simplicity, on interpretation of the OGCM open ocean circulation (i.e. 50 km off shore) and only considered the first coastal zone that the particles came across when advected backwards – and discarded the later ones. Calculating connectivity by including every coastal zone the particle travelled through – rather than just the first one – did not change the results (Fig. S5).

Another potentially important factor that our results could be sensitive to is the influence of decadal variability considering that our region of interest is under the influence of the IPO. To evaluate the sensitivity of the EIE connectivity, the Lagrangian experiment was reproduced within two other phases of the IPO: for the decades 1968–1977 (negative IPO) and 1990–1999 (positive IPO). Patterns remain unchanged with highest frequency found for the South American coast within 20°S–40°S. However, stronger connectivity is observed in that band with 53% and 60% of land particles for 1968–77 and 1990–99 experiments, respectively. The results also indicate a rather weak sensitivity to the period over which the statistics is performed (Figs. 4c, S7) indicating that the climatological currents contribute the most to the connectivity pattern documented in this study.

## Discussion and Conclusions

We examined the exposure to plastic debris waste of the remote island Easter Island (Rapa Nui) and its ecoregion (EIE) where important contamination of its marine ecosystems occurs, and identified upstream ocean circulation pathways that connect to potential coastal and oceanic hotspots of marine plastic waste generation within or bordering the South Pacific basin. We modeled pathways for both neutrally- and positively-buoyant plastic debris.

**Figure 5:**
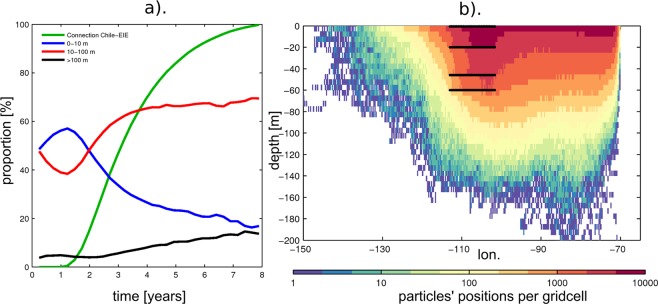
Focus on particles connecting Easter Island Ecoregion to Chilean coast (See Fig. 3e for footprint). (**a**) proportion of particles reaching Chilean coast over time (green). For each timestep, the total particles still travelling is decomposed into proportion of particles travelling within 0–10 m (blue), 10–100 m (red) and >100 m depth (black). (**b**) Illustration of particles distribution with depth using position within 30 °S–20 °S (within dashed lines in Fig. 3e). 5-daily positions are binned in 0.25° by 5 m gridcells. Black lines show particle release locations.

While the model formulation (i.e. resolution, forcing, period) appears to have a marginal impact on our results (Fig. 4), the sensitivity analysis to the buoyancy of the particles through the consideration of either the 2D or 3D velocity fields raises interesting issues. In particular, although there is little sensitivity to the latitude at which the particles originate, the connectivity appears strongest for buoyant plastics with 97.5% of Lagrangian particles (deployed and advected backwards in time around Easter Island and coming into contact with land) originating from the Pacific eastern coastline (Table 1). Considering the case of neutrally-buoyant “virtual” model particles, this connectivity is weaker with 77.1% of particles from the eastern Pacific. A new pathway with the Malay Archipelago appears wherefrom 12.5% of connecting particles originate. These are predominantly carried through the Equatorial Under Current at depth of ~200 m and through the anti-cyclonic surface (0–70 m) circulation of the gyre. Three-dimensional pathways show that a significant part of incoming particles travels within depth of 10–100 m, which approximately corresponds to the ranges of mean depth of the mixed layer in the South Eastern Pacific. In the case of connecting particles between the EIE and South America, 50–70% of particles travel within these depths, with the majority of the remaining 30–50% within the surface 0–10 m layer **(**Fig. 5). If plastic debris behaves like the neutrally-buoyant “virtual” particles modelled here, a significant proportion may be travelling below the surface and not appear in sampling surveys that have focused on the surface. The focus here is to examine 3D pathways, so far unconsidered, relative to 2D ones and not to make a full account of all potential 2D plastic debris pathways e.g. at the surface, subsurface, including wind-induced mixing or Stoke’s drift. For instance, the impact of Stoke’s drift on material dispersal can be important, as was shown in the Southern Ocean^54^, and therefore could also affect plastic drifting pathways at the surface in the Pacific basin^55,56^. However, this is beyond the scope of this study but should be considered for future work.

We find the ecoregion strongly connected to the eastern side of the basin and particularly with the coastal region between 20°S and 40°S consisting of suitable places where to implement management strategies that could mitigate plastic contamination of the EIE. The Chilean coastal region shows that in the case of neutrally-buoyant plastic experiment, between 47.5% and 60% (of the total particles contacting coastlines within the South Pacific) has origin within 20°S and 40°S of the South American continent, depending on the decadal period chosen. Beside its strength, this pathway has the shortest connectivity timescales of the entire basin with the 10^th^ percentile of fastest particles taking less than 1.6 years to cross over. This connectivity is even stronger in the 2D experiment representative of floating debris (Table 1). This region corresponds to a region of Chile with the highest population density (including large coastal cities and the capital Santiago) and contains several major rivers^57^, representing both important sources of ocean plastic inputs. This part of Chile could therefore represent an appropriate choice for implementing/reinforcing plastic management strategies. However, it is difficult to establish quantities of plastic waste entering the ocean for a given region. Chile presents a low estimate of mismanaged plastics within 50 km of the coast^50^ mainly due to a smaller production and more efficient management (7% of mismanaged plastic equivalent to ~46,000 tons; ref. ^50^; World Bank Indicators) than other Pacific South-American countries such as Peru (25%, 377,000 tons) and Ecuador (30%, 211,000 tons), which have weaker connectivity with 20.6% and 5.1%, respectively (Table 1). Yet, mismanaged plastic for this area may be under-estimated as it does not include the metropolitan region of Santiago de Chile, located ~100 km from shore, hosting 8 million people and generating 43% of the national waste^58^. Mismanaged litter from inland communities reaches the coastal zone of Chile via rivers^57,59^.

This study shows that Easter Island is also strongly connected to marine regions where intense industrial fishing is taking place. This strong connection with fishing zones is underlined by the high percentages of recognizable litter stranding on Easter Island that can be attributed to the industrial fishery operating in the South Pacific^26,30^. A fast and direct pathway connects the Peru Fishing Zone (FZ-P) with the EIE, 18.5% of the 3D-experiment particle deployed pass through this fishing zone with the fastest 10% taking less than 1.4 years (Figs. 1 and 4). In the 2D experiment – more relevant to predominantly-buoyant fisheries waste – this proportion decreases to 15.5% as surface pathways lie further south (Fig. S4). For comparison to land-source connectivity, 22.5%, and 42% of all particles deployed within the EIE (including those that reach land and those that do not) connect with Chile in the 3D and 2D experiments, respectively (Fig. 3).

This higher connectivity with the continent contrasts with the low proportion of recognizable continental litter items stranding on Easter Island^26,30^, which suggests that continental plastic litter either does not reach the EIE or does so in an already degraded state. Similar results were recently reported for the oceanic Inaccessible Island in the South Atlantic, where most recognizable litter also seems to have oceanic sources originating from shipping and fisheries^60^.

The proximity of the gyre could explain the large proportions of plastic fragments observed — as plastic in the gyre is already in a degraded form — but cannot explain why so little distinguishable land-based litter is observed on the island’s beaches. If continental litter reaches the region only in an advanced state of degradation, this means it has travelled very slowly. However, this study shows the majority of plastic originating from South American coast reaches the island in a 1.8–2.8 year timescale – taking 25th and 75th quantile. A potential explanation may be that un-degraded plastic waste get removed from the surface within this timescale, through processes that accelerate its degradation such as ingestion by wildlife^61^ – that return plastic to the environment in smaller pieces – or through processes that slow down its dispersal to EIE e.g. sinking induced by bio-fouling exposing objects to subsurface slower currents. Removal of particles due to fouling-induced sinking from the surface can occur for debris as large as cm-sized particles^14^, oscillatory vertical behavior^16^ may decrease the speed at which debris travel to the EIE. Another possibility is that continental plastic waste is predominately entering oceans in an already degraded state due to time spent in rivers, sewage systems or rocky coastal zones (e.g., ref. ^62^).

Higher state of fragmentation could also be explained by land-waste being less robust to the marine environment than purpose-built fishery equipment. Also, more-buoyant fisheries products are constrained to the surface and may travel faster than smaller, fragmented terrestrial plastic waste. High representation of fishery items in floating litter has also been observed elsewhere, e.g., in the North Pacific gyre^7,60^. The high proportion of recognizable fisheries plastic waste might also indicate that these come from the less intense industrial fishery operations in the immediate vicinity of the EIE, i.e. from sources closer to the target area.

These considerations highlight that the industrial fishery operating in the high seas of the South Pacific and the other areas of the high seas must be regulated not only with respect to their extractive activities but also for their solid waste disposal, which contribute significantly to the plastic contamination not only of the open ocean but also the coastal zones via circulation connectivity (e.g., ref. ^63^). Ongoing UN negotiations on establishing a legally binding instrument on the governance of the High Seas provide a unique opportunity to address downstream impacts of the high seas activities on the coastal zones, including pollution by plastic debris. This study contributes to a growing evidence of the direct exposure of the coastal zone and its ecosystem services to the activities on the high seas and the harmful impacts they produce. Implementation of recent laws, such as the Chilean law 20920 about waste management^64^, which aims (among others) to reduce the use of non-recyclable plastics, is another step in this direction.

It is important to consider our results in a context of the climate change. Modification of the ocean currents (e.g., ref. ^65^) and resulting patterns of connectivity (e.g., ref. ^66^) are among the key climatic stressors of the marine environment. As the main boundary currents change their strength and position, connectivity patterns of the remote ocean islands to the main land sources of plastic pollution are likely to change in the next decades. Thus, when developing a long-term mitigation strategies, it is important to stress the need to carry out re-evaluations of (i) the connectivity patterns as impacts of the climate change on the ocean circulations become more pronounced, and (ii) the models providing future projections of the ocean environment improve.

During recent decades, Chile and other South American countries along the Pacific coast have established large oceanic Marine Protected Areas (MPAs), e.g., the Galapagos Marine Reserve (Ecuador), the Motu Motiru Hiva Marine Park (Chile), or the Nazca-Desventuradas and Mar de Juan Fernández Marine Parks (Chile)^67,68^. Plastic litter is one of the main threats to these marine ecosystems with sources often located long distances away from the respective MPAs (e.g.,^26^). Herein, we assess the exposure of a remote pristine marine ecosystem, the Easter Island Ecoregion (EIE), to plastic-waste pollution from both land- and marine-based sources. We identify ocean circulation pathways that transport such plastic litter to the EIE at basin scale, by examining 3D and surface 2D circulation and, as such, we consider transport of both predominantly macro-sized floating plastic and more neutrally-buoyant micro-sized plastic debris, respectively, and therefore present a more comprehensive view of incoming pathways to the EIE than 2D pathways alone. Coastal Chile and the intense commercial fishing region just offshore of the Peru Exclusive Economic Zone represent important plastic debris pollution threats for the EIE and present fast and strong connectivity pathways. Successful protection of MPAs from plastic debris contamination through effective plastic waste mitigation will require rigorous measures being applied to open ocean fisheries as well as to land-based sources. Our case study suggests that efficient strategies for protecting oceanic marine reserves requires the collaboration of all sectors of society.

## Supplementary information

Supplementary information 
