# Supplementary material for: In search for the sources of plastic marine litter that contaminates the Easter Island Ecoregion
Source: Sci Rep. 2019 Dec 23;9:19662. doi: 10.1038/s41598-019-56012-x (PMC6927966; doi:10.1038/s41598-019-56012-x)
Supplement: Supplementary file 1 — Supplementary information [file 41598_2019_56012_MOESM1_ESM.docx]

In search for the sources of plastic marine litter that contaminates the Easter Island Ecoregion

**Supplementary Material**

Simon Jan van Gennip^1,2, 3, 4^ *., Boris Dewitte^1,2,3,5^, Véronique Garçon^3^, Martin Thiel^1,2,5^, Ekaterina Popova^6^, Yann Drillet^4^, Marcel Ramos^1,2,7^, Beatriz Yannicelli^1,2^, Luis Bravo^1,2^, Nicolas Ory^1,2,8^, Guillermo Luna-Jorquera^1,2^, Carlos F. Gaymer^1,2^

^1^ Millennium Nucleus for Ecology and Sustainable Management of Oceanic Island (ESMOI), Coquimbo, Chile

^2^ Departamento de Biología Marina, Universidad Católica del Norte (UCN), Coquimbo, Chile

^3^ Laboratoire d'Etudes en Géophysique et Océanographie Spatiales (LEGOS), Toulouse, France

^4^ Mercator-Océan International (MOI), Ramonville, France

^5^ Centro de Estudios Avanzados en Zonas Áridas (CEAZA), Coquimbo, Chile

^6^ National Oceanography Centre (NOC), Southampton, UK

^7^ Centro de Innovación Acuicola Aquapacífico, Coquimbo, Chile

^8^Cluster of Excellence "The Future Ocean", GEOMAR Helmholtz Centre of Ocean Research Kiel, Marine Ecology Department, Düsternbrooker Weg 20, 24105, Kiel, Germany.


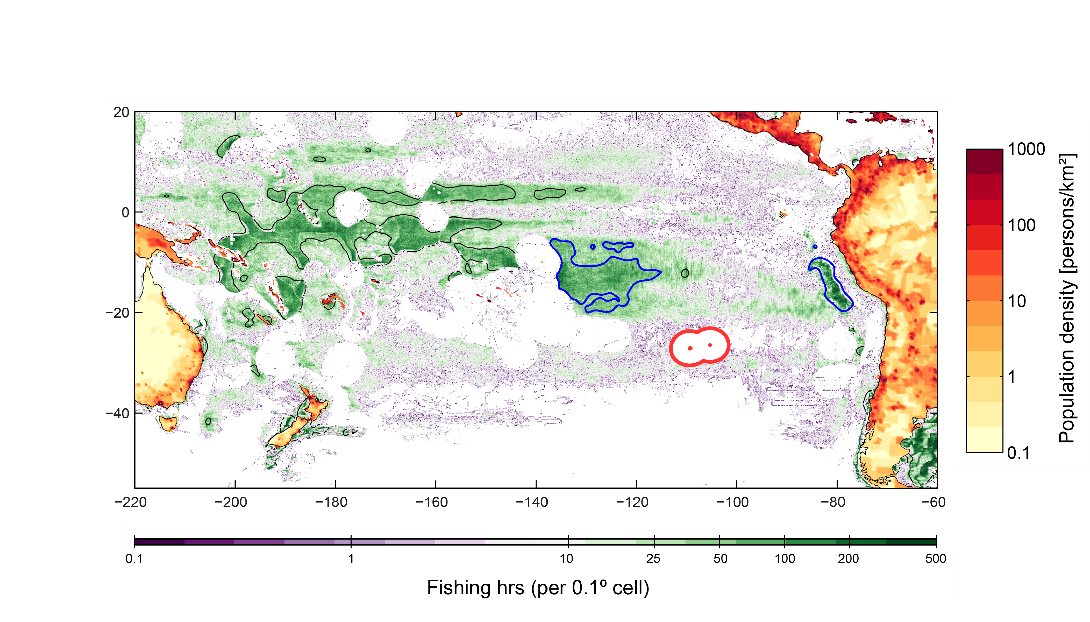


**Figure S1:** Population density and fishing effort for the Pacific region. Connectivity of Easter Island and its Ecoregion (red contour) is calculated with coastal region of the South Pacific basin and also with two intense commercial fishing zones (blue contours: Central Pacific Fishing zone, FZ-CP; and Peru Fishing Zone ,FZ-P) so to identify the potential threat of exposure to plastic litter that gets generated in these regions. Fishing effort corresponds to the cumulative number of hours fished in a given gridcell (resolution of 0.1degrees) between 2012-2016. The contours correspond to cumulated fishing of 50 hours. Fishing effort and population density datasets are obtained from globalfishingwatch.org/ and the Center for International Earth Science Information Network, Columbia University United Nations, Food Agriculture Programme, and Centro Internacional de Agricultura Tropical.


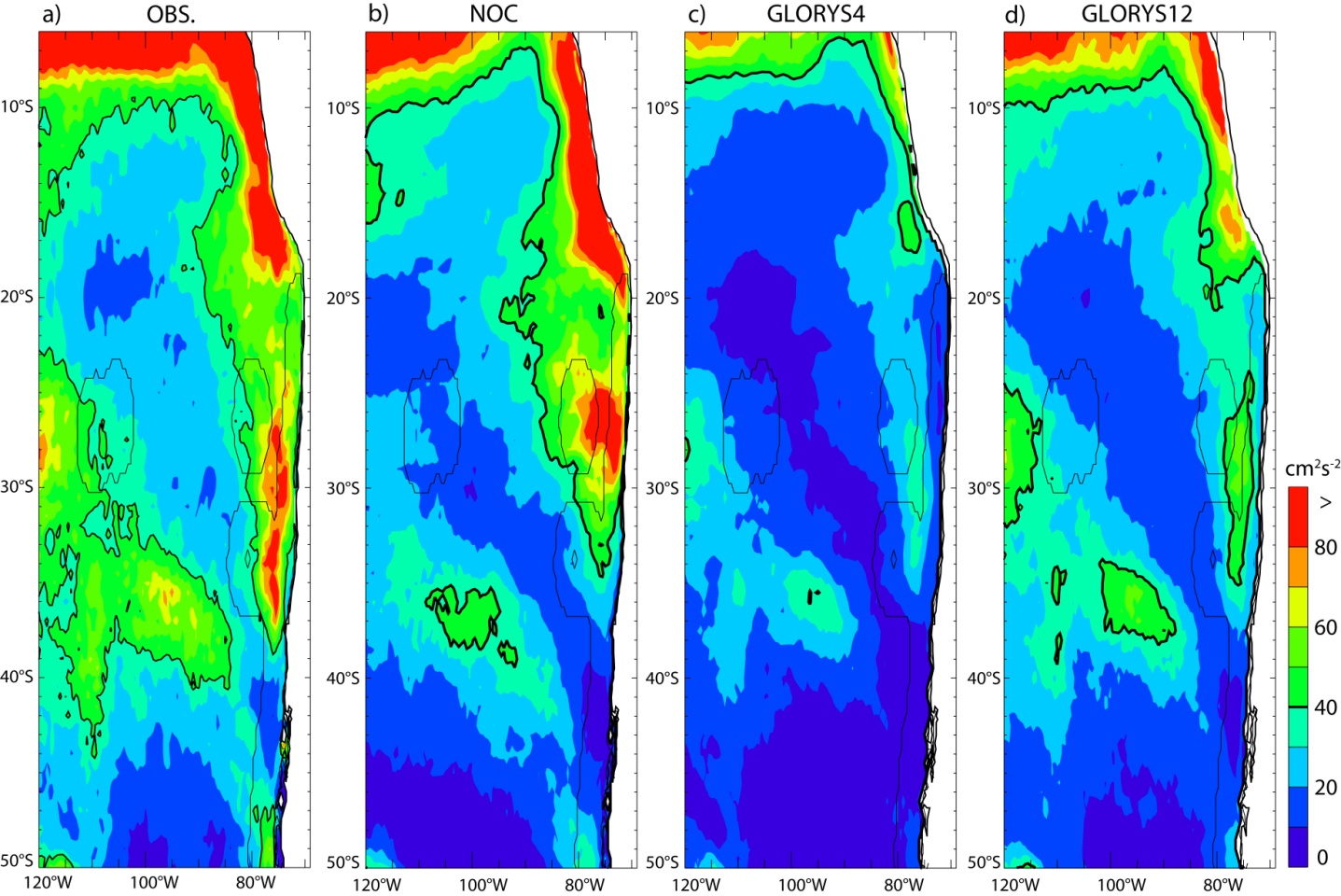


**Figure S2** : Mean eddy kinetic energy (EKE) associated to geostrophic surface current for (a) altimeter data, (b) NOC), (c) GLORYS4 and (d) GLORYS12 for the period 1993-2015. Data are from the GlobCurrent products of Ifremer. All data consist in 5-day mean averages and anomalies to derive the mean EKE are relative to the mean seasonal cycle calculated over the period 1993-2014. The thin black line on all the maps indicates the Exclusive Economic Zone of Chile.

**S3: Example of connectivity with the intense fishing region off the coast of Peru and Central Pacific.**


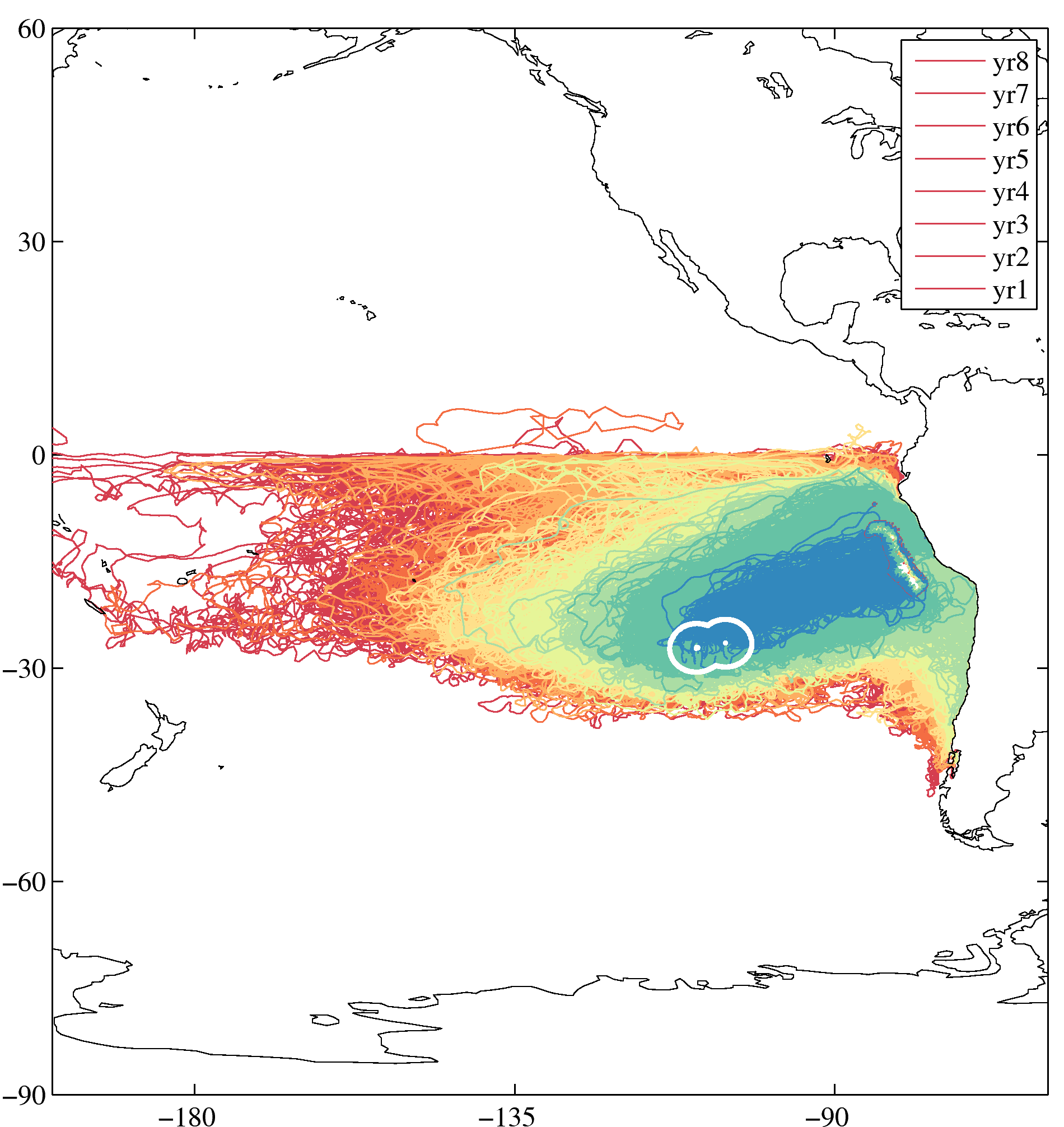

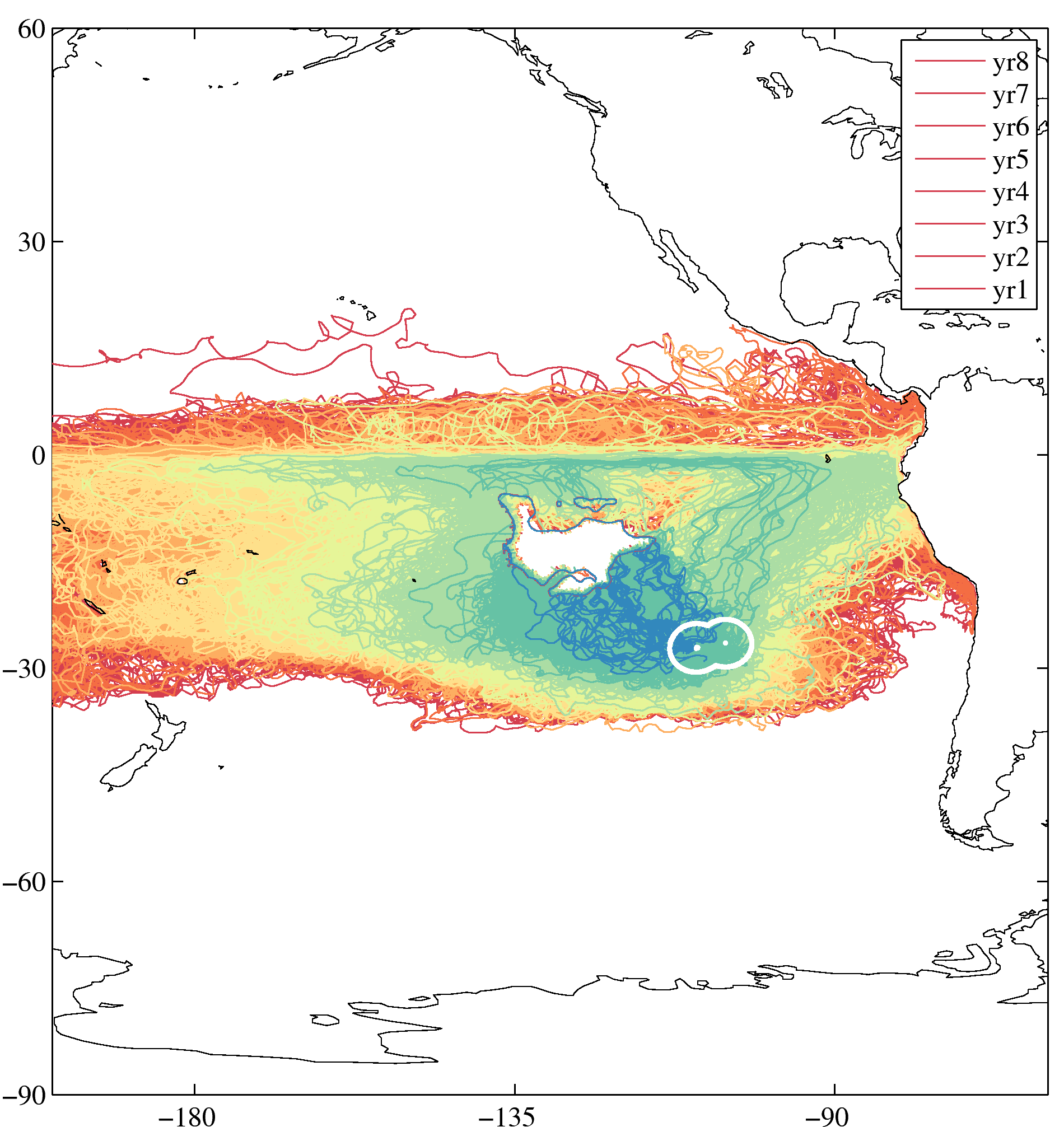


Figure S3: Trajectories deployed within Easter Island Exclusive Economic Zone (EEZ, white contour), backward in time, in the 3D oceanic flow and connecting to the intense fishing region situated off the coast of Peru (left) and to its northwest in central south Pacific (right). Trajectories' colour corresponds to the time, rounded down, taken by the particles to connect the two sites, e.g. blue for trajectories of particles taking between 1 and 2 years, dark green between 2 and 3 years, etc.

**S4: Connectivity Footprints and time distribution between Easter Island and intense fishing region within Central Pacific, off the coast of Peru, and coastal Chile.**


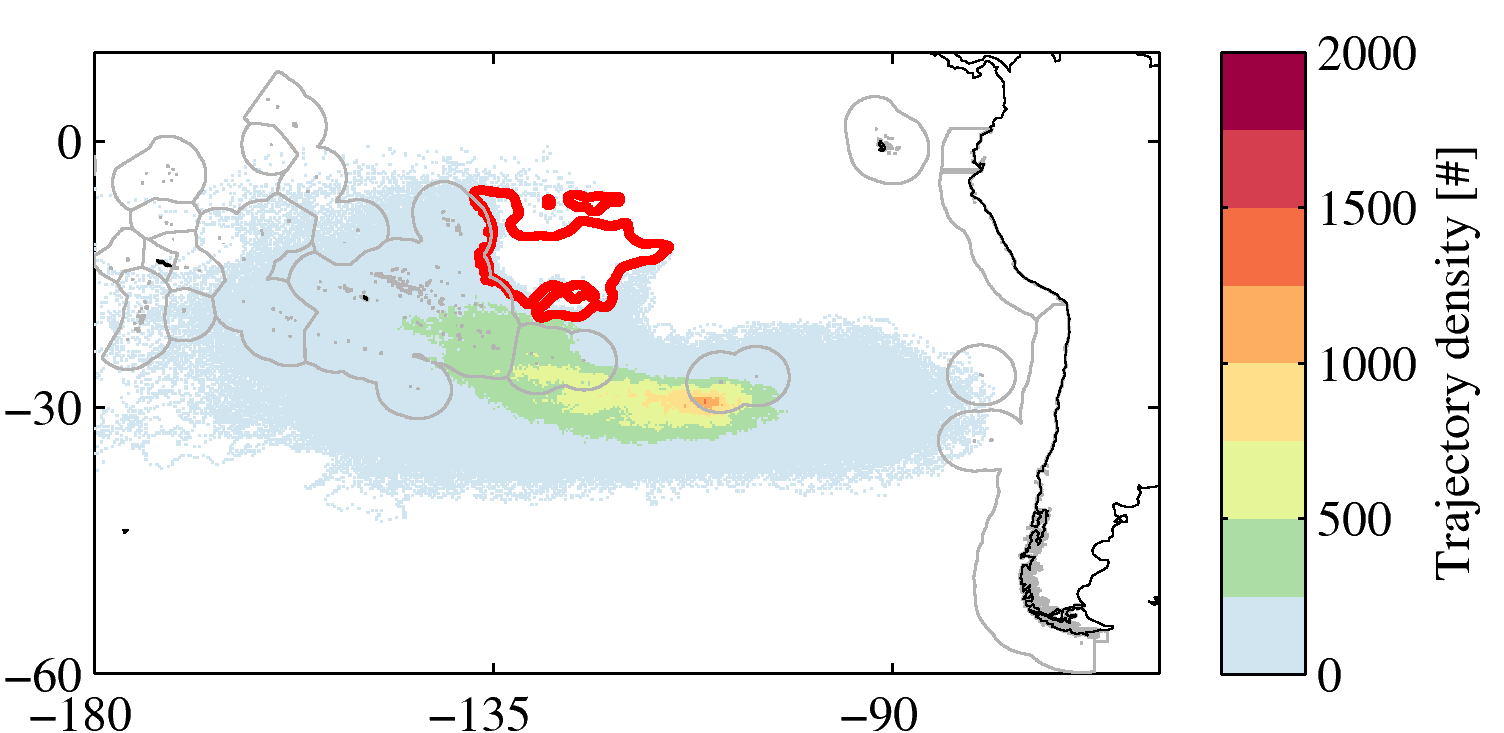

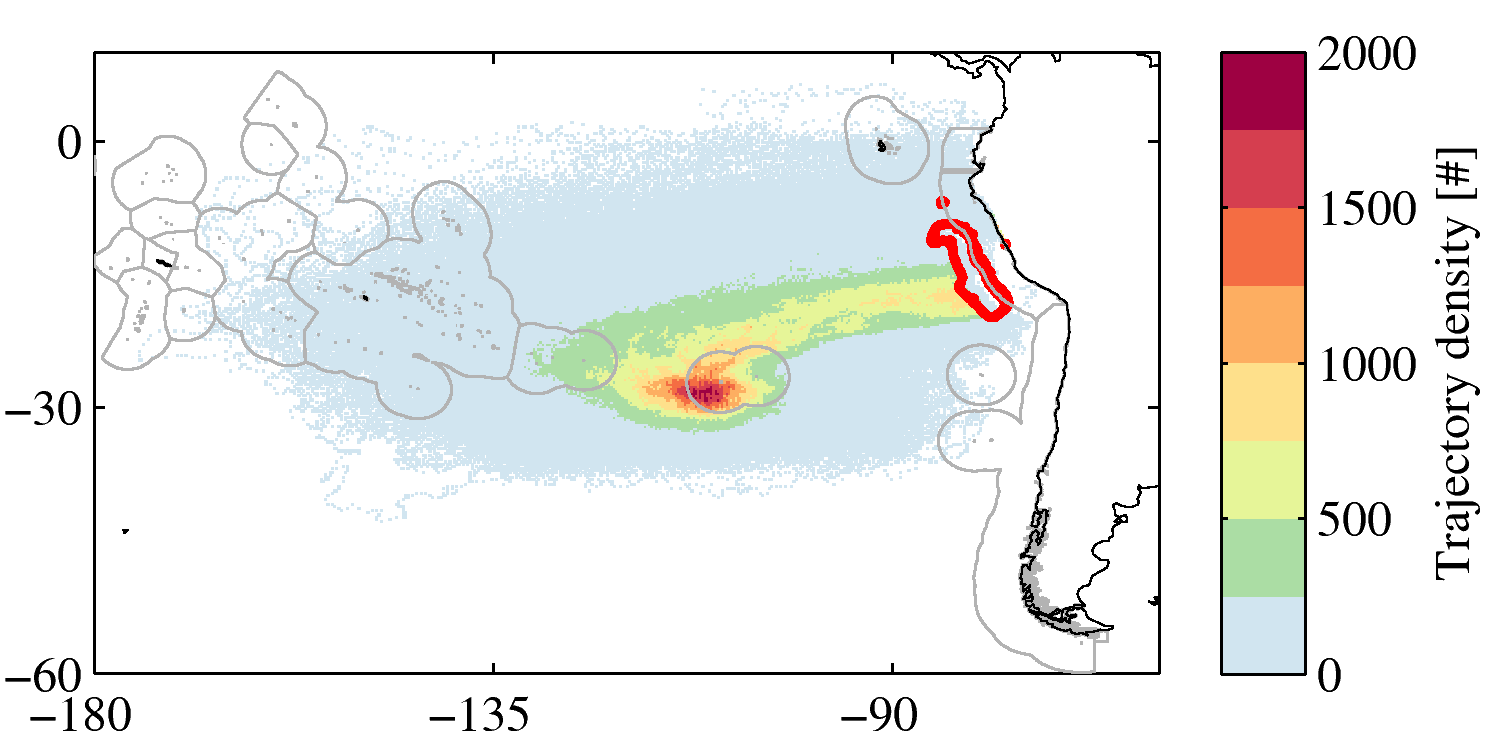

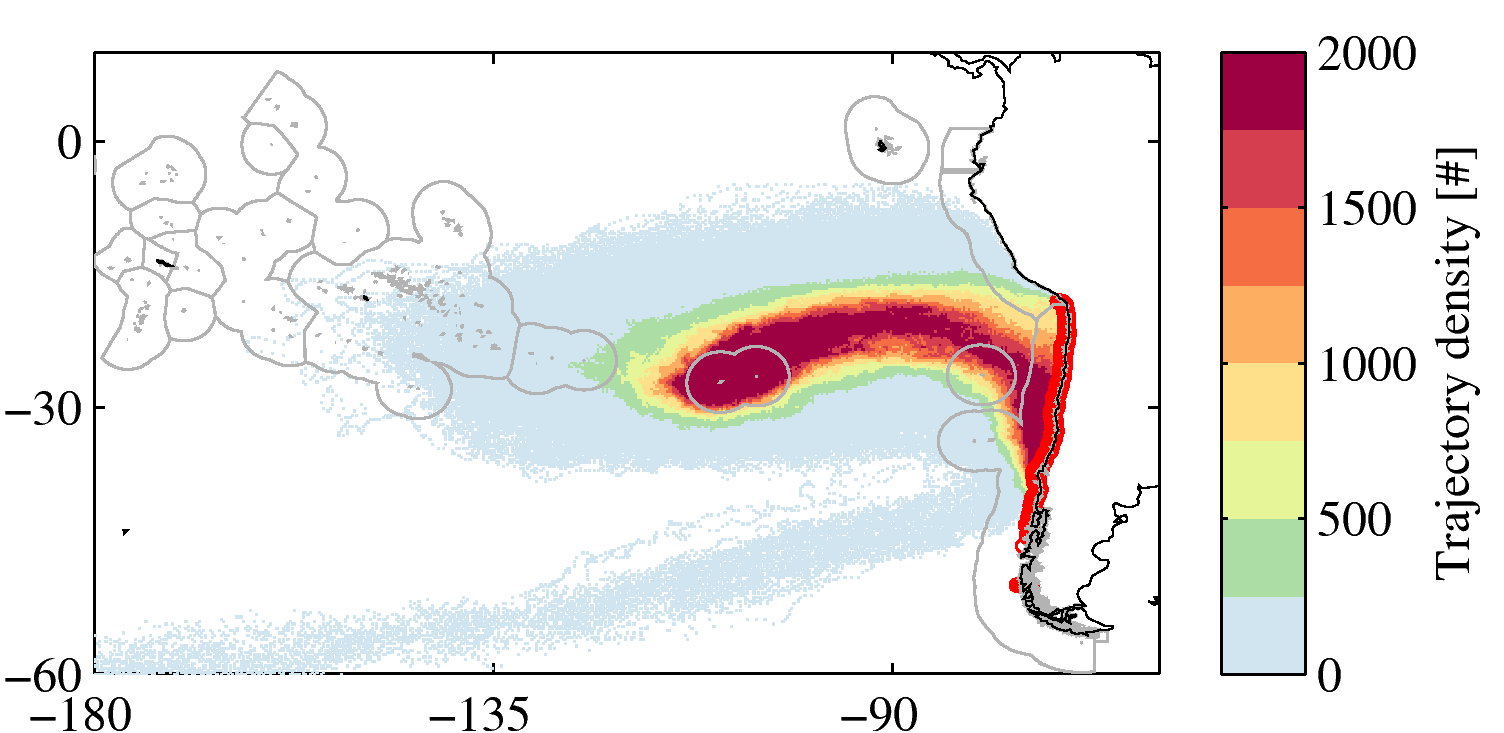

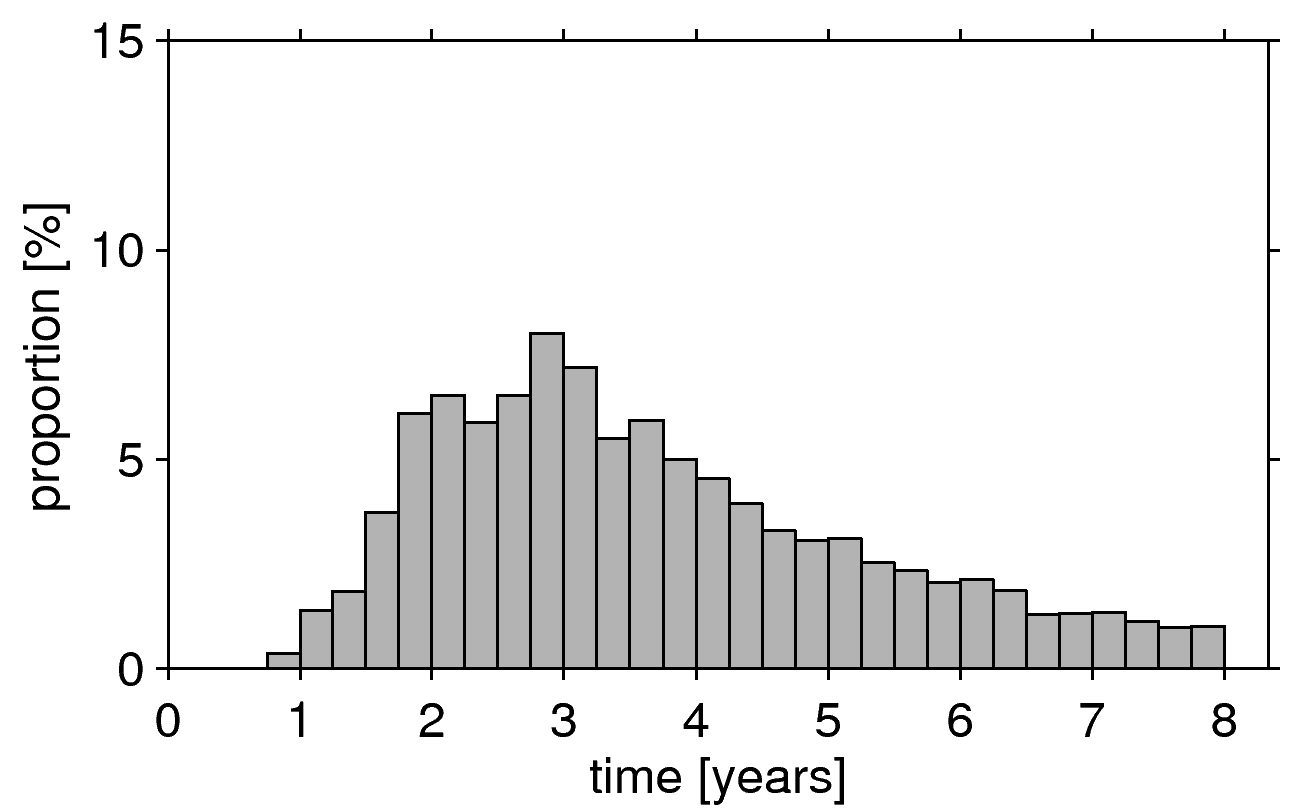

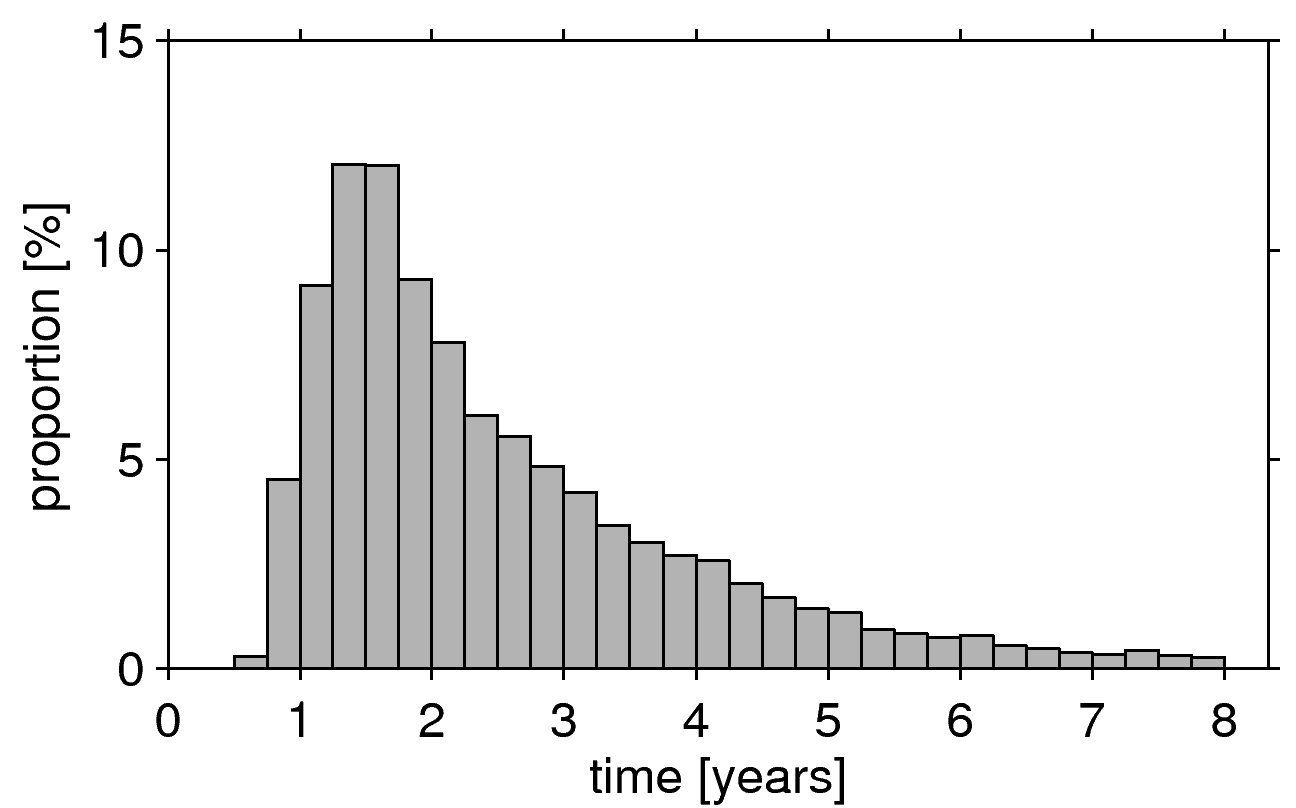

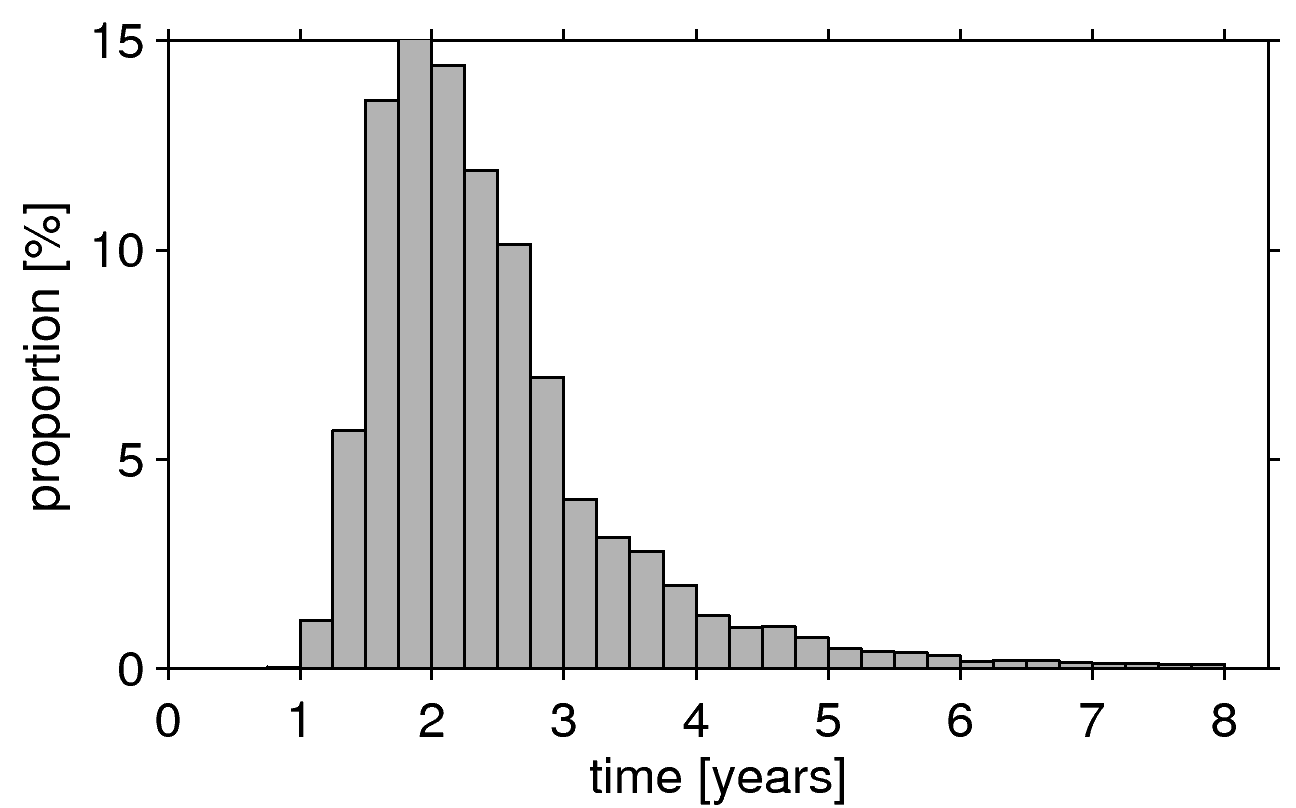


Figure S4: Same as figure 3 but for the experiment carried out in the surface flow.

**S5: Sensitivity to method:**
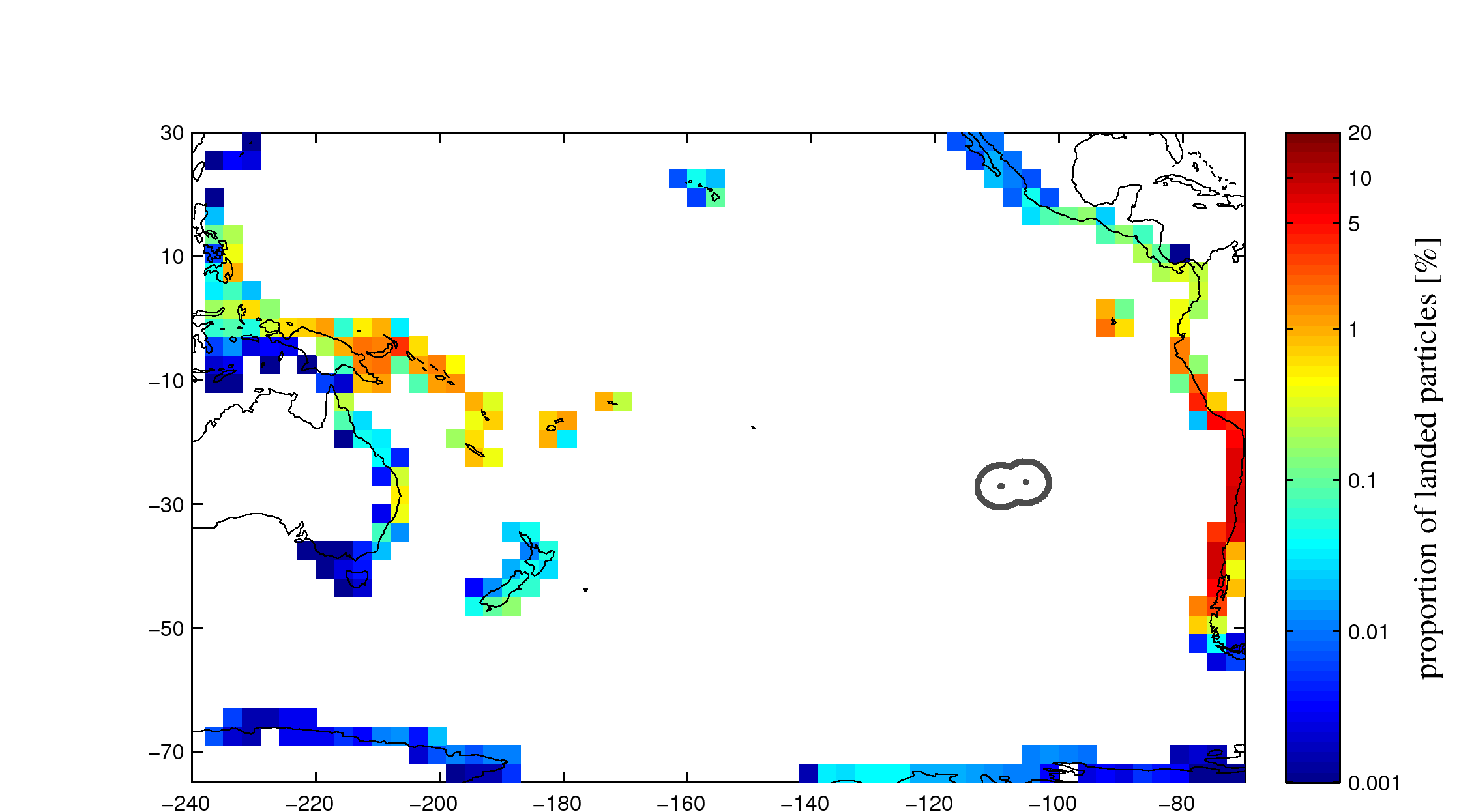

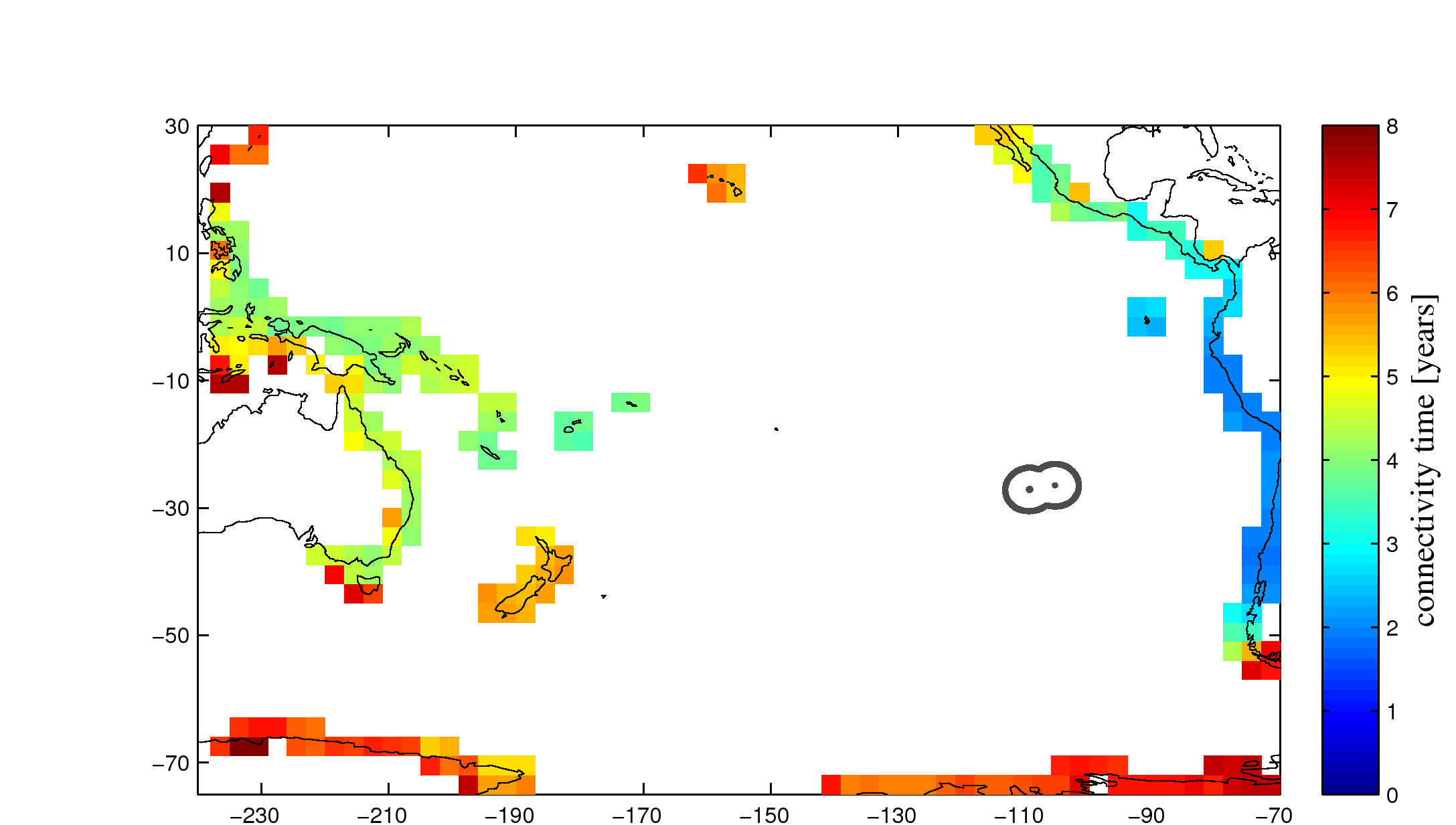


Figure S5: Same as figure 2, but considering all of the coastal regions that the particles travelled through and not only just the last one. Note the logarithmic scale. With this method the sum of proportion of each gridcell exceeds 100% as particles can connect to multiple coastal sections.

**S6: Decadal variability in the statistics**


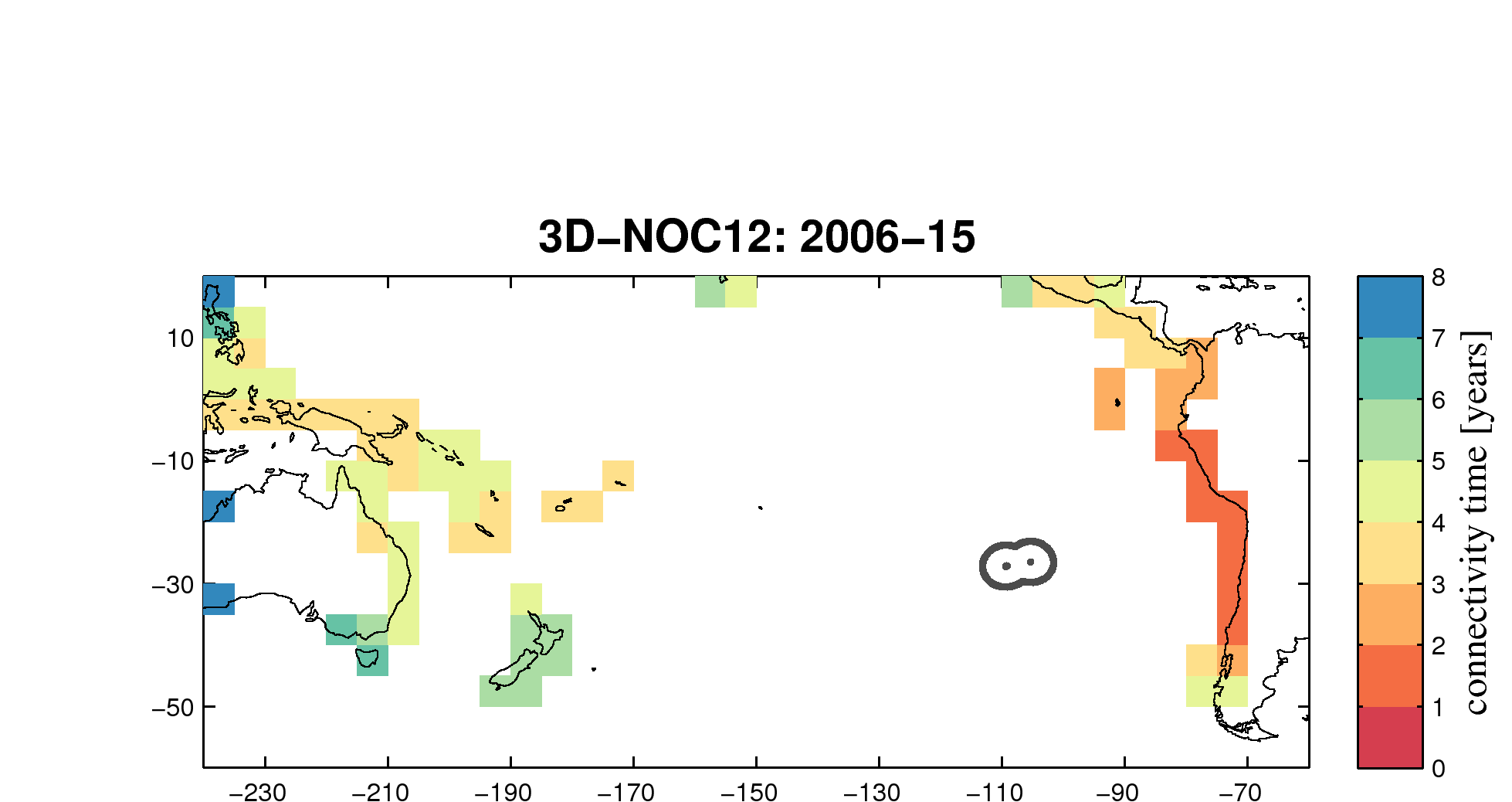

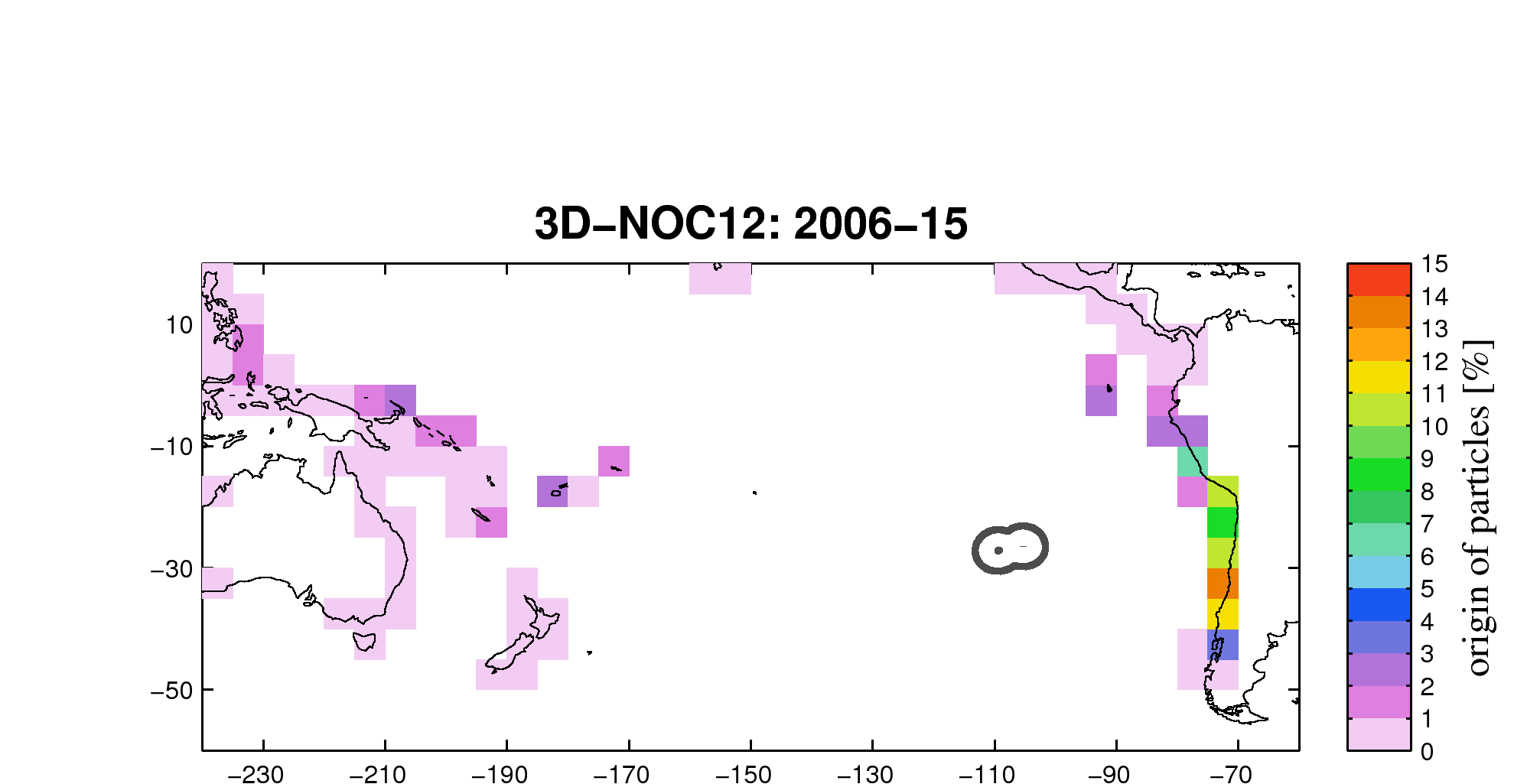

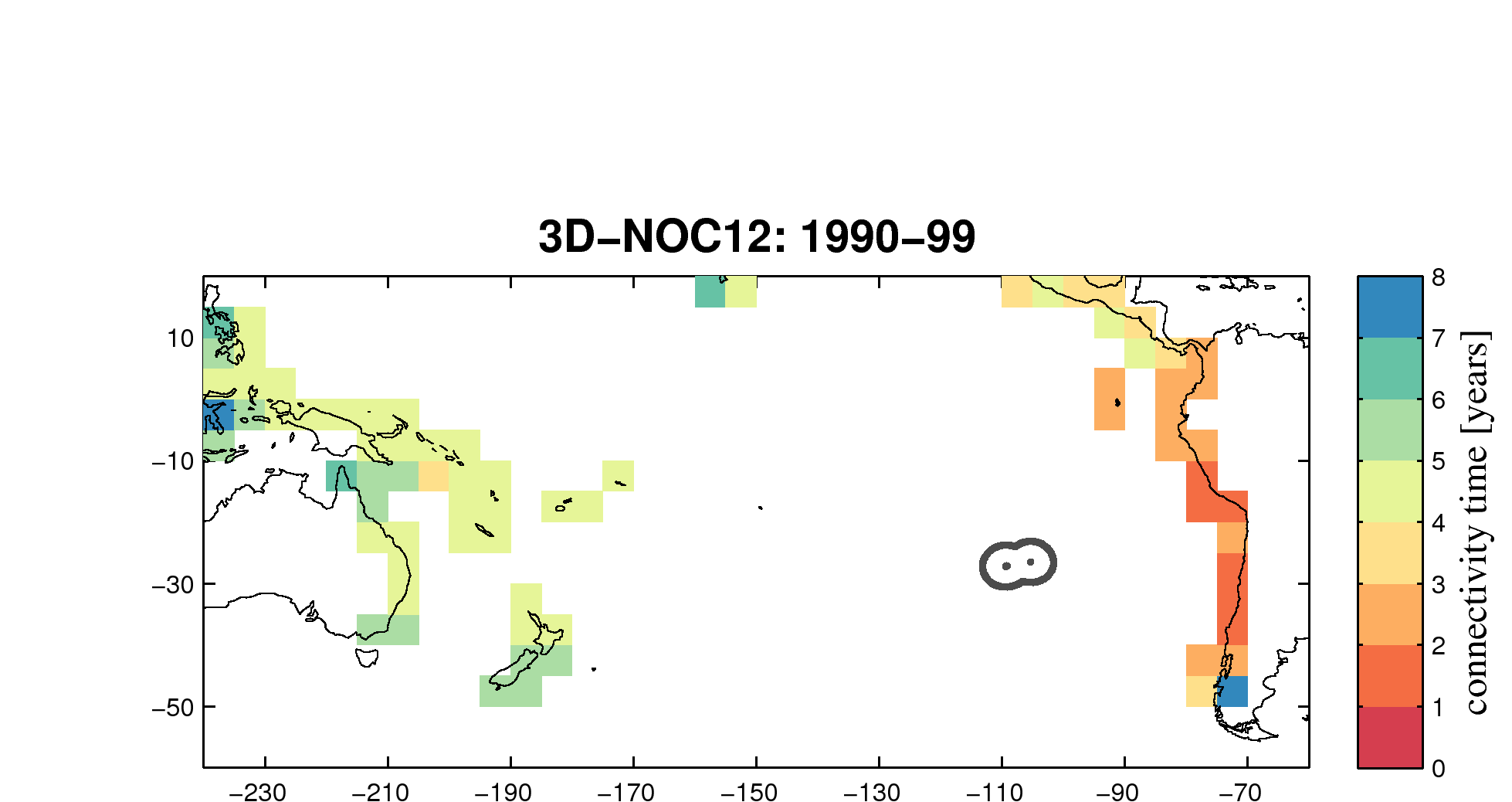

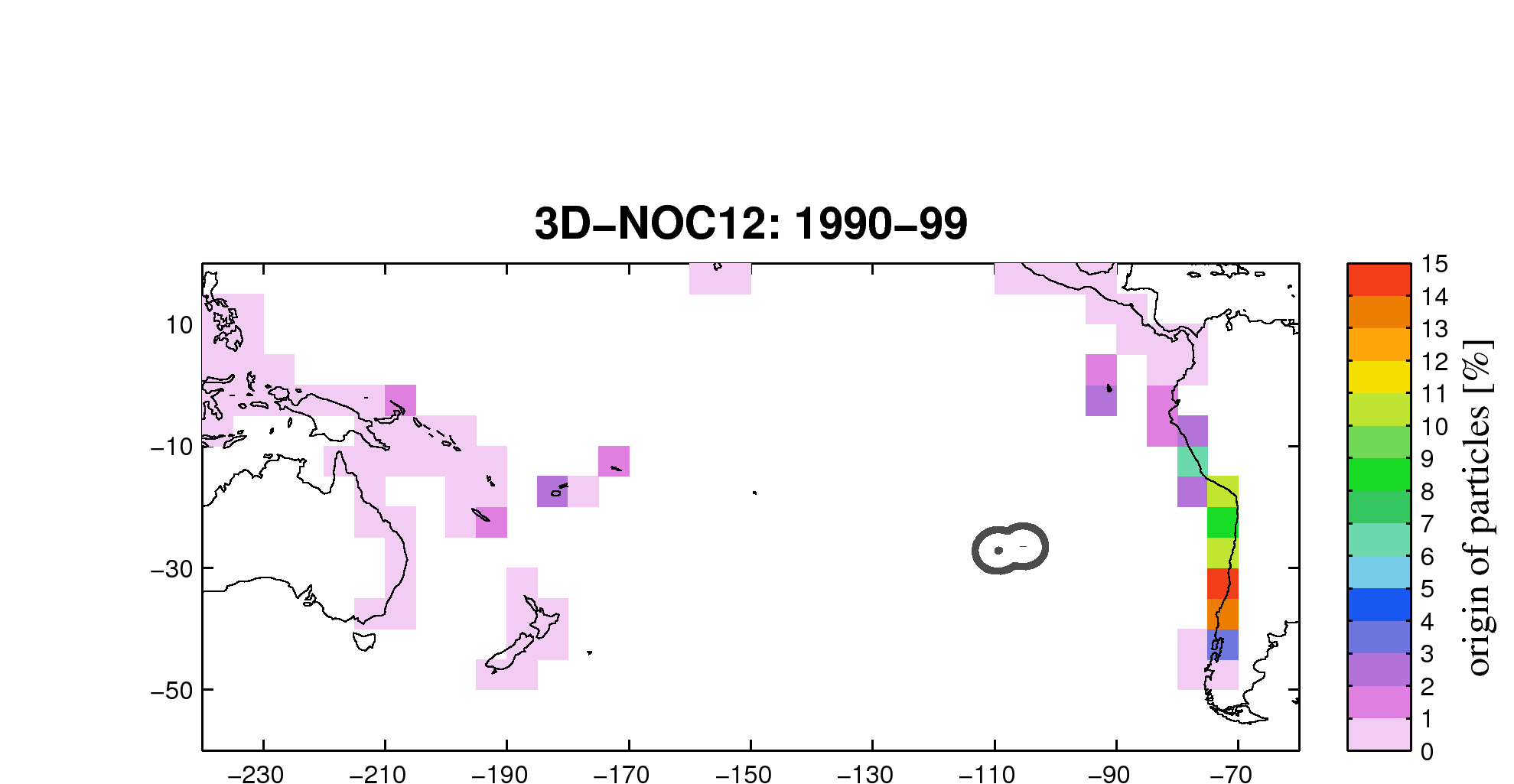

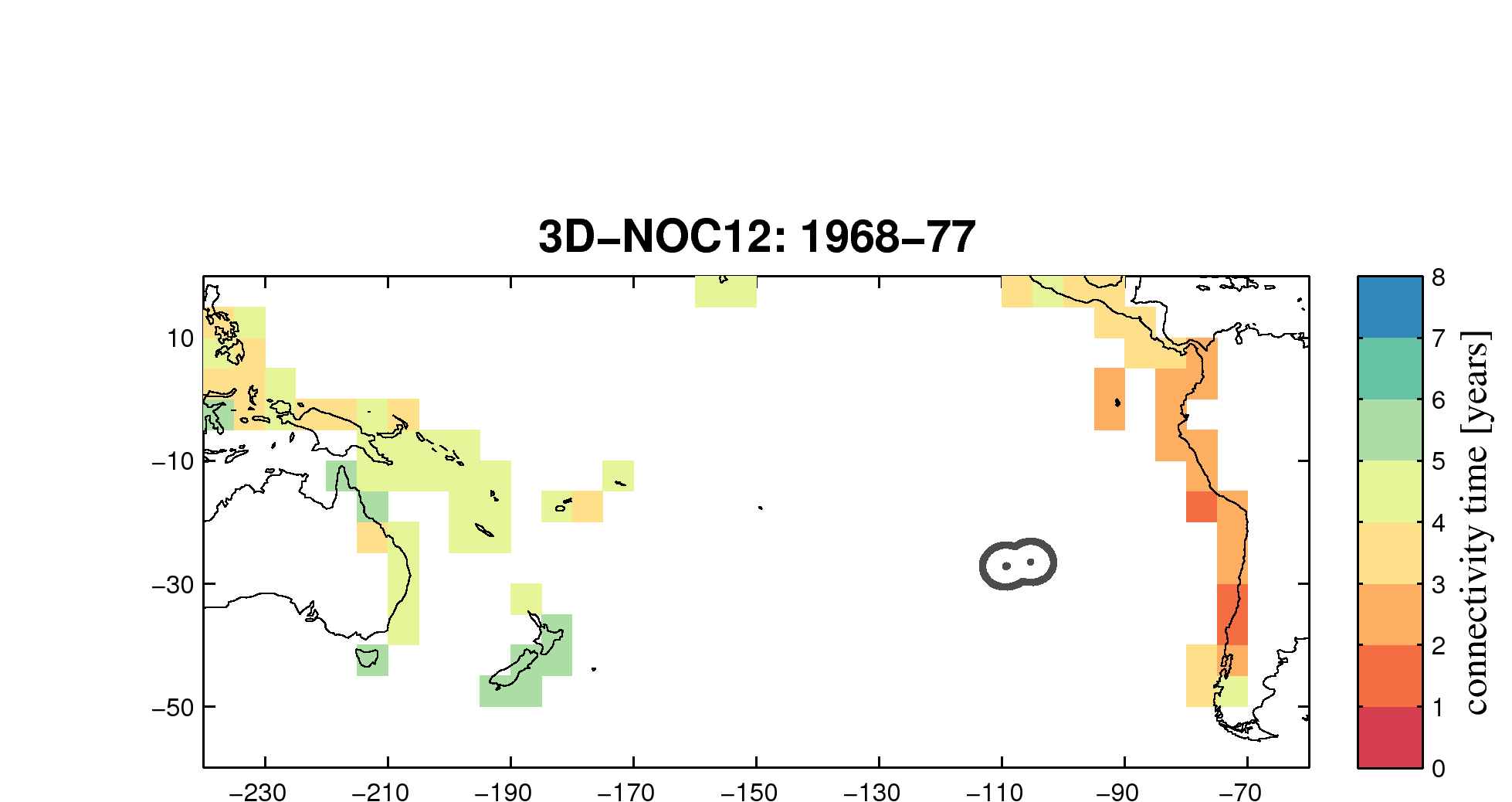

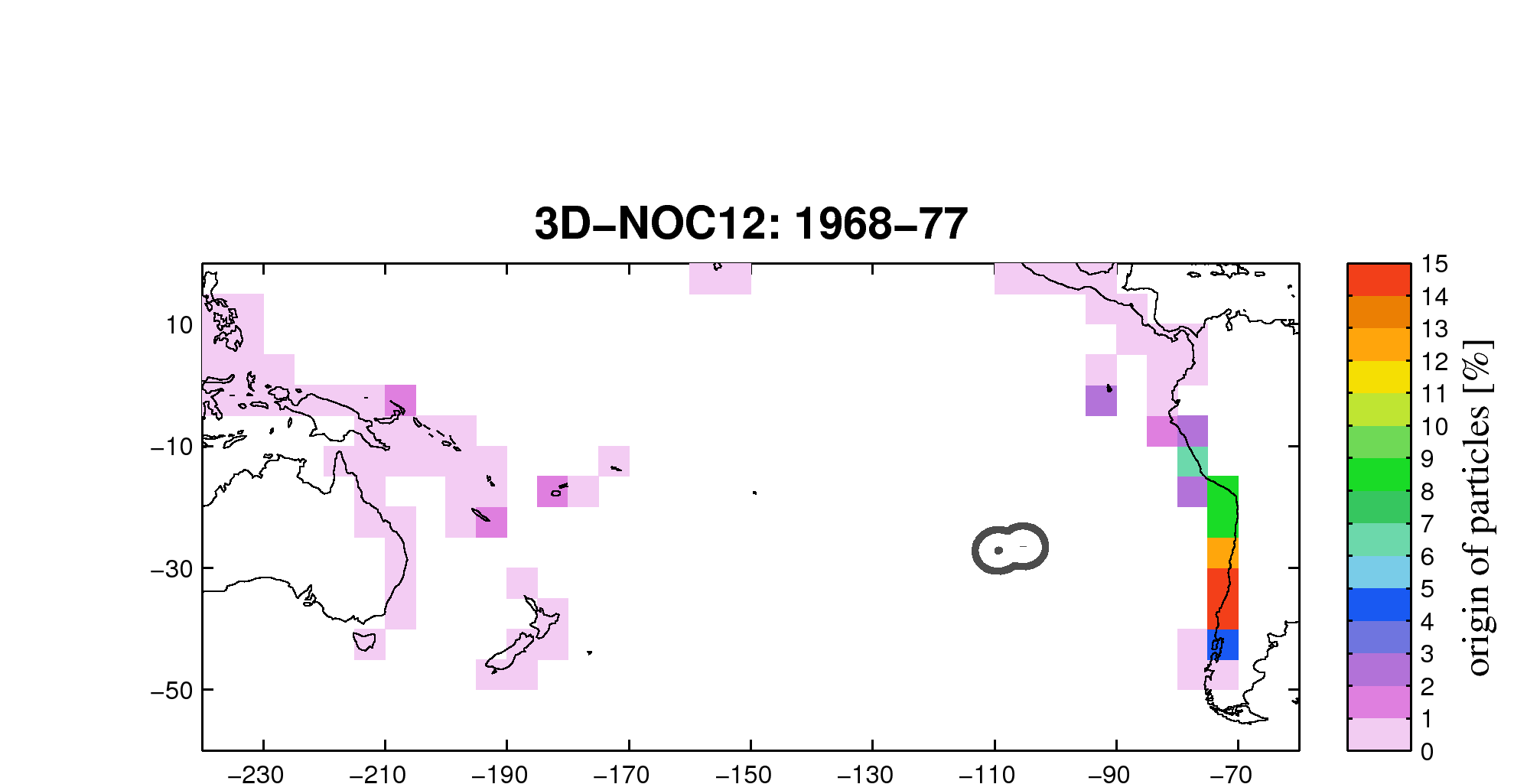


Figure S6: Connectivity strength (top) and time (bottom) of Easter Island with coastal regions of the Pacific basin obtained from experiments carried out in 2006-15 (left), 1990-99 (middle) and 1968-77 (right) and corresponding to different period of the Interdecadal Pacific oscillations.

**S7: sensitivity to period of study: focus on South American coast**


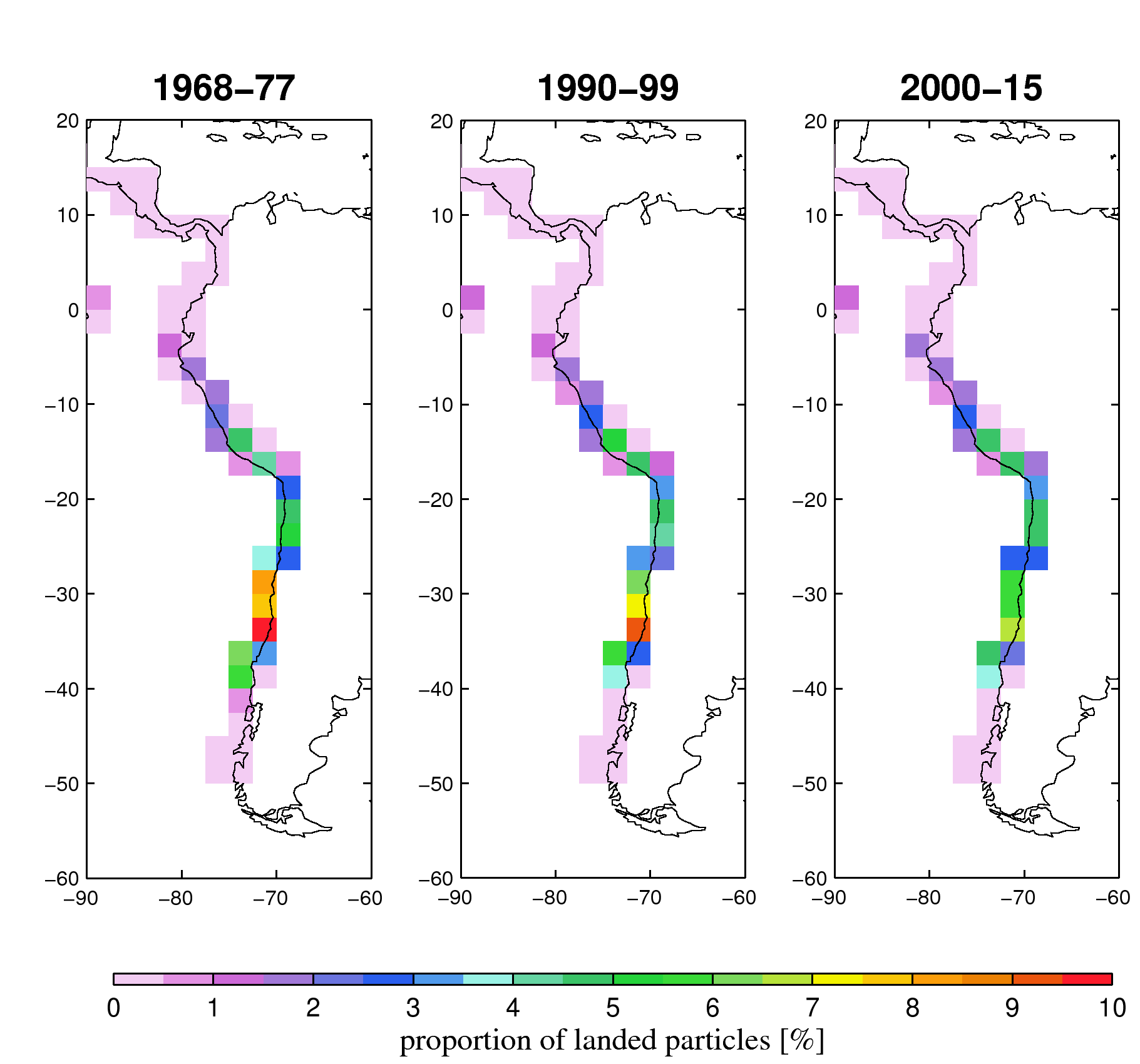

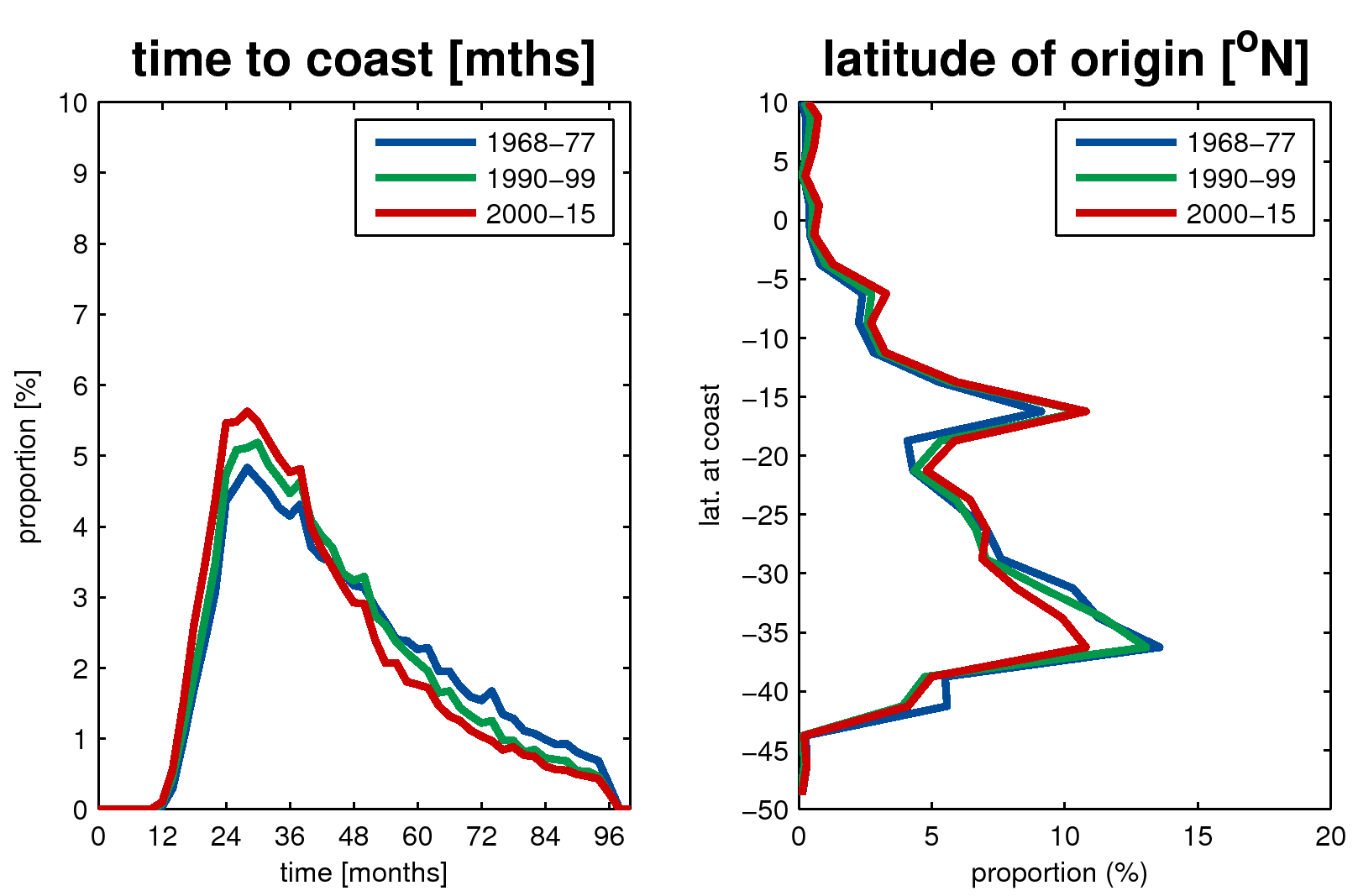


Figure S7: Same as figure S6 but focusing on the South American coast. Note the change in the size of resolution (2.5 by 2.5 degrees). The plot on the right displays the proportions of particles for each period of study as a function of latitude.

**S8 Sensitivity to turbulence:**


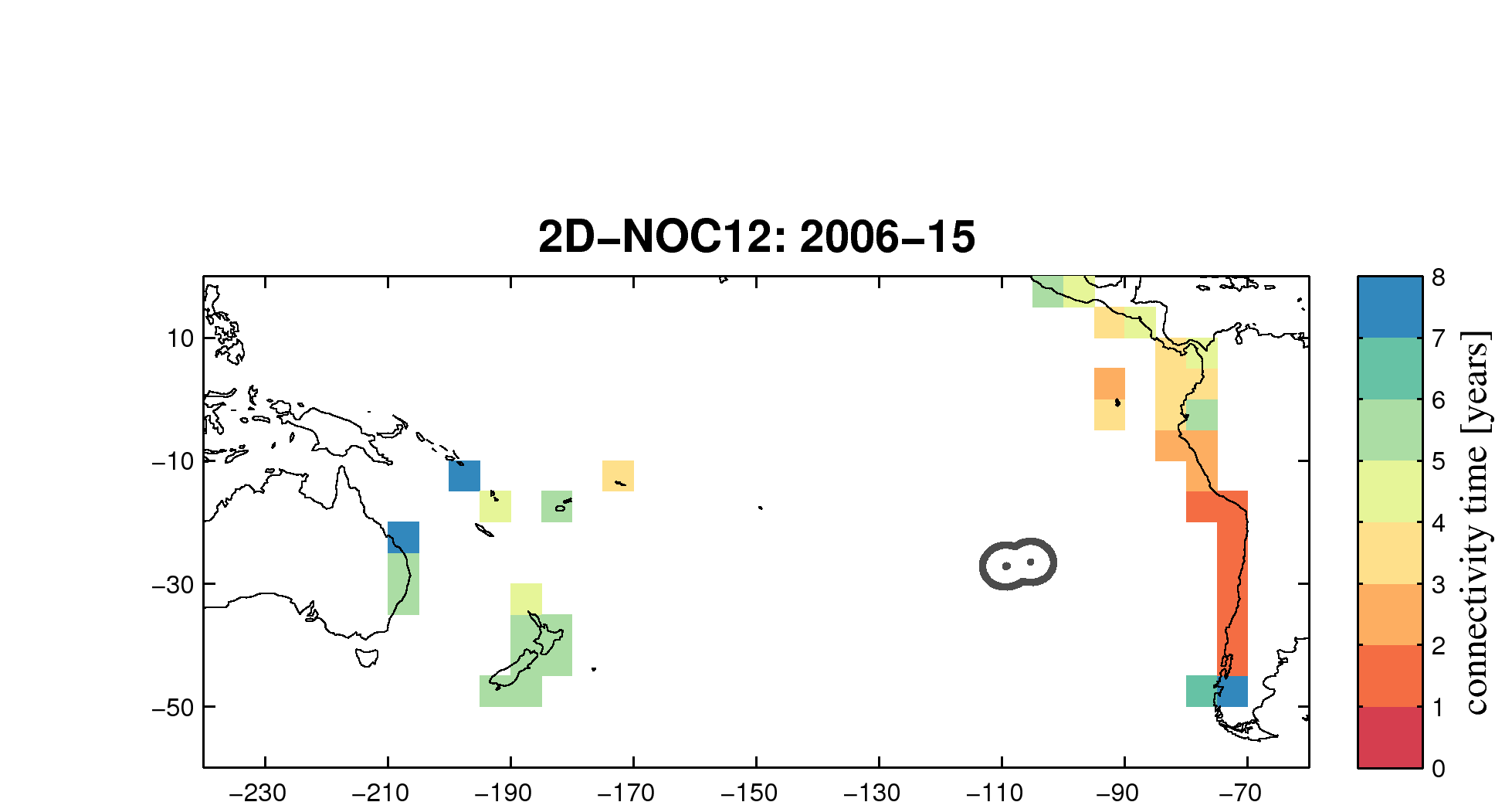

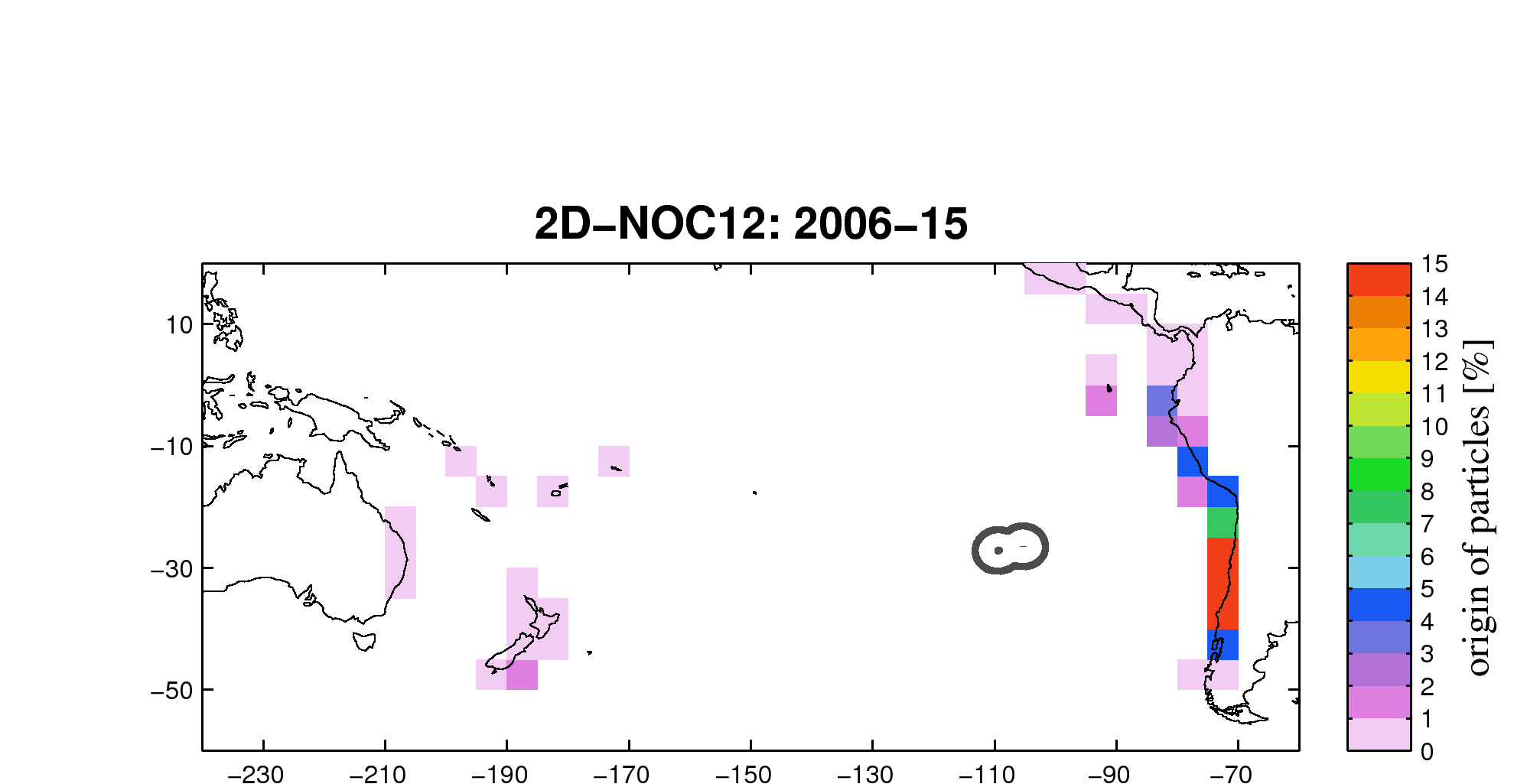

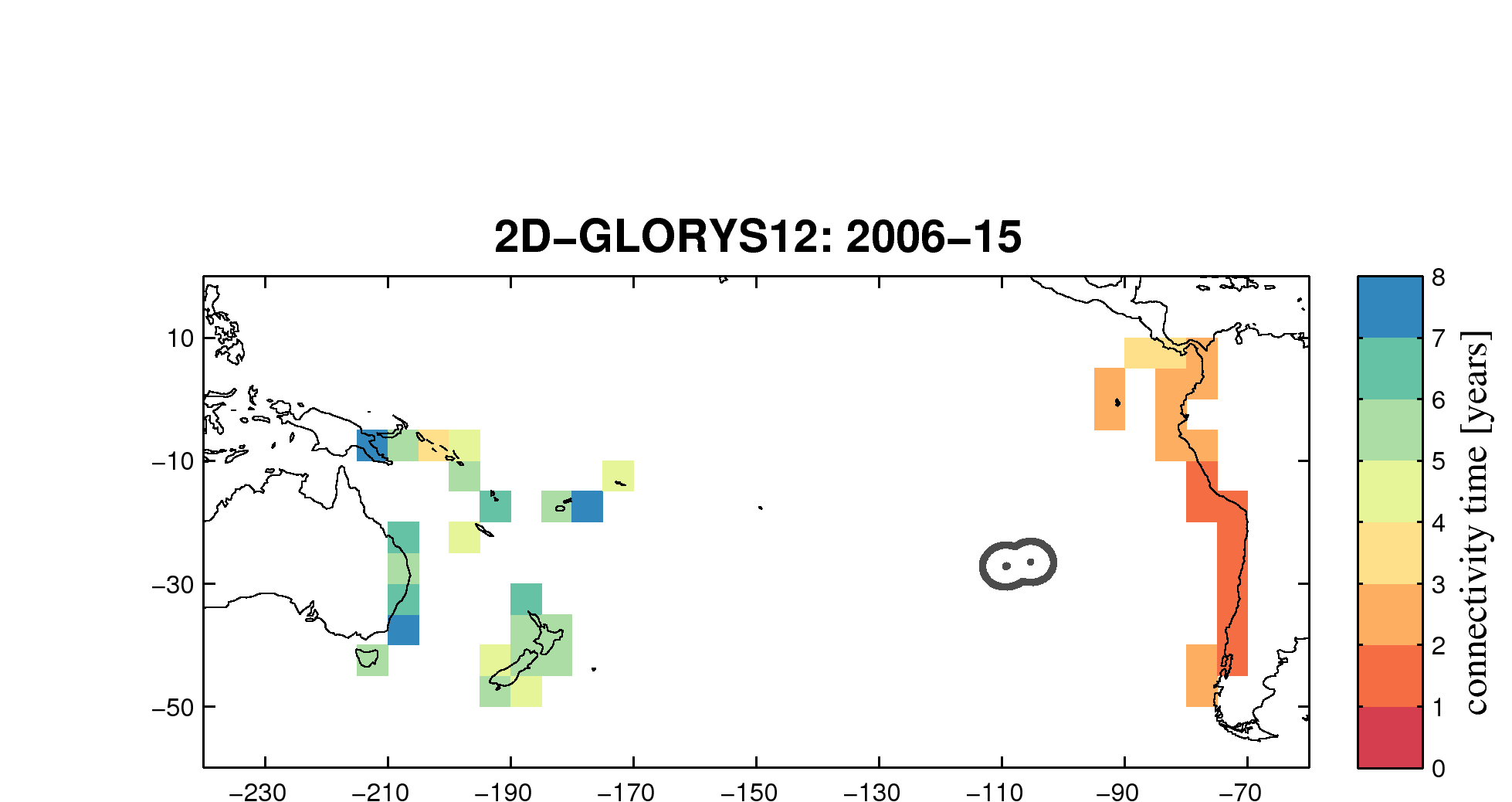

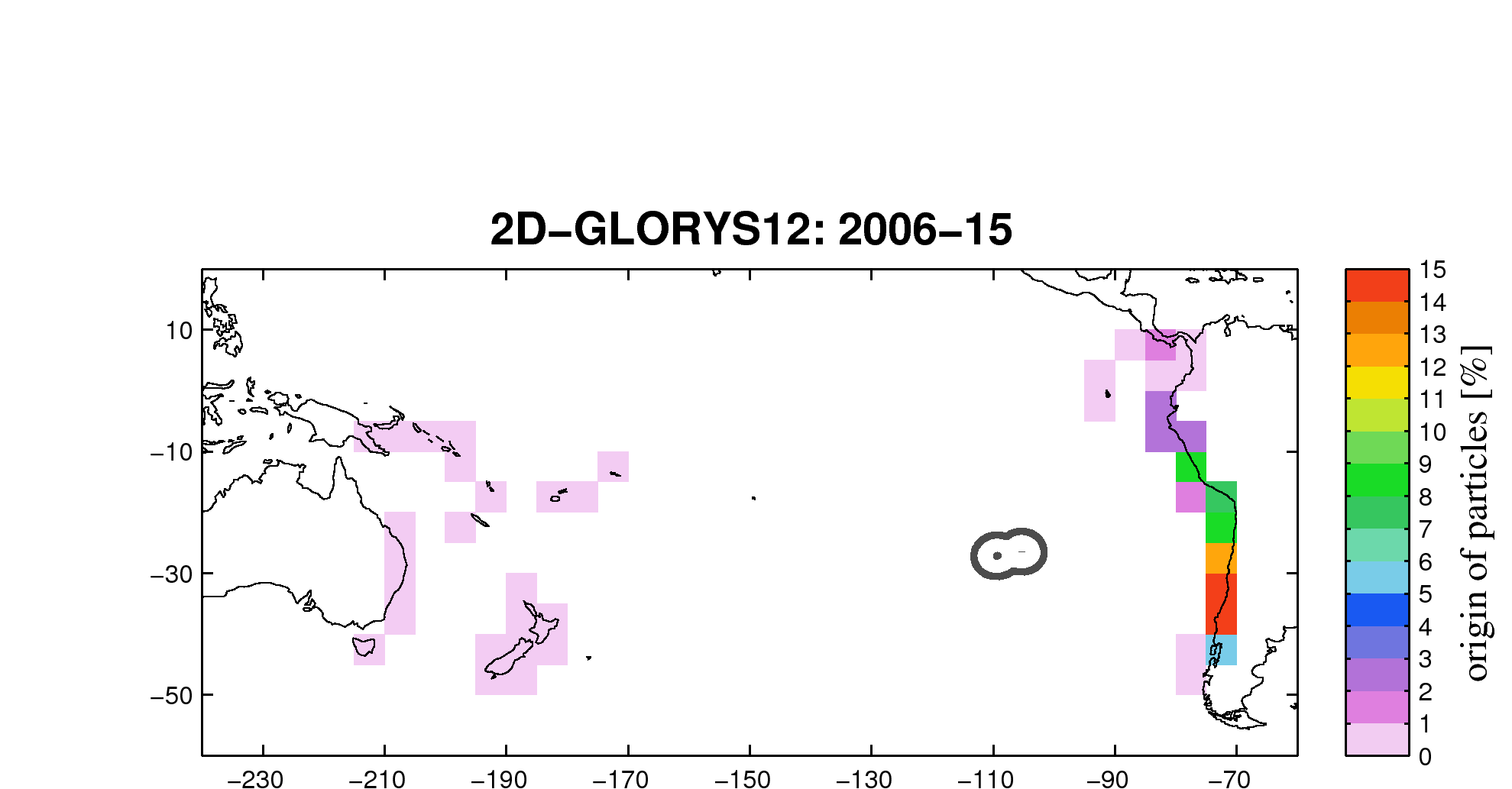

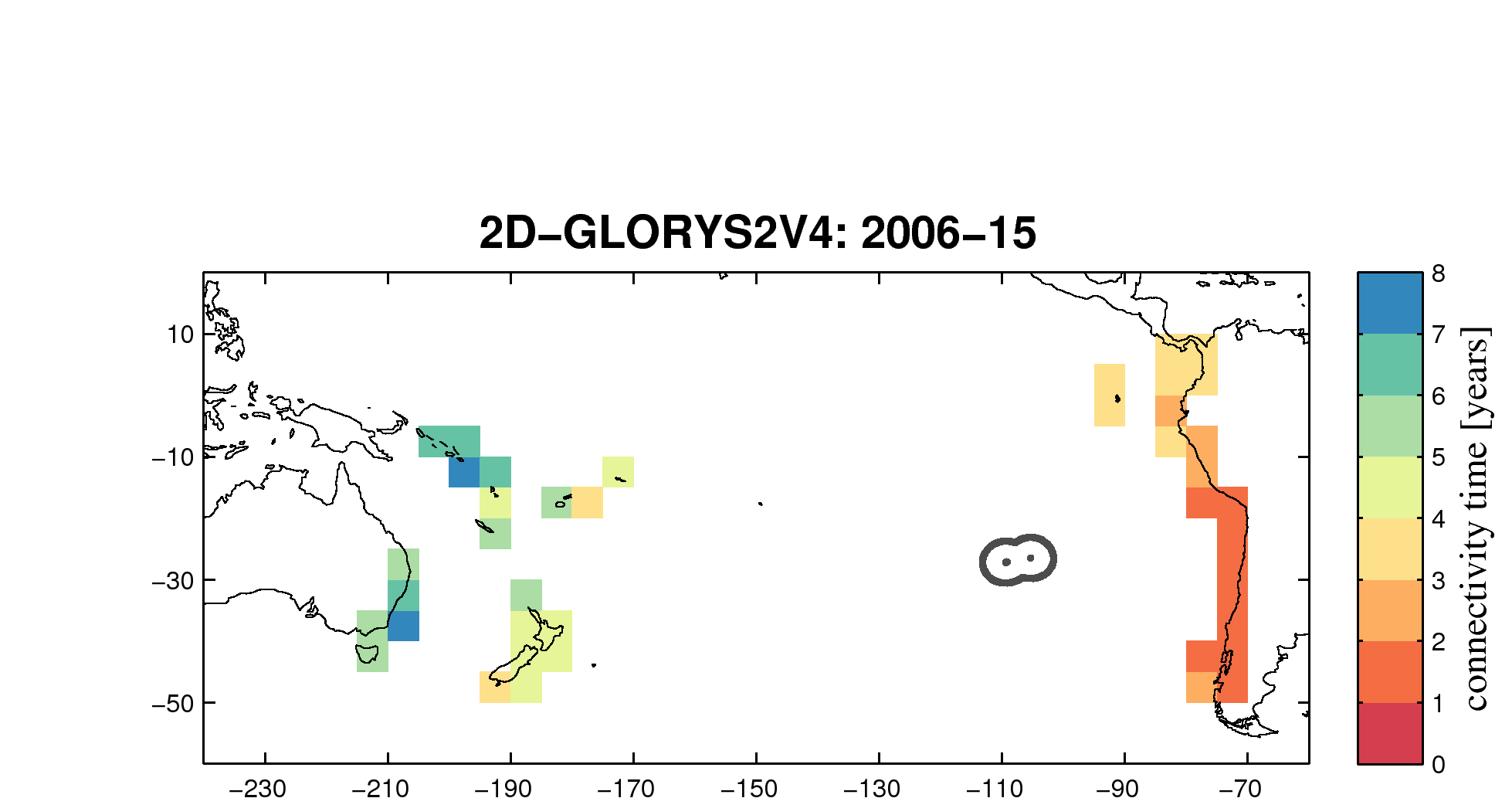

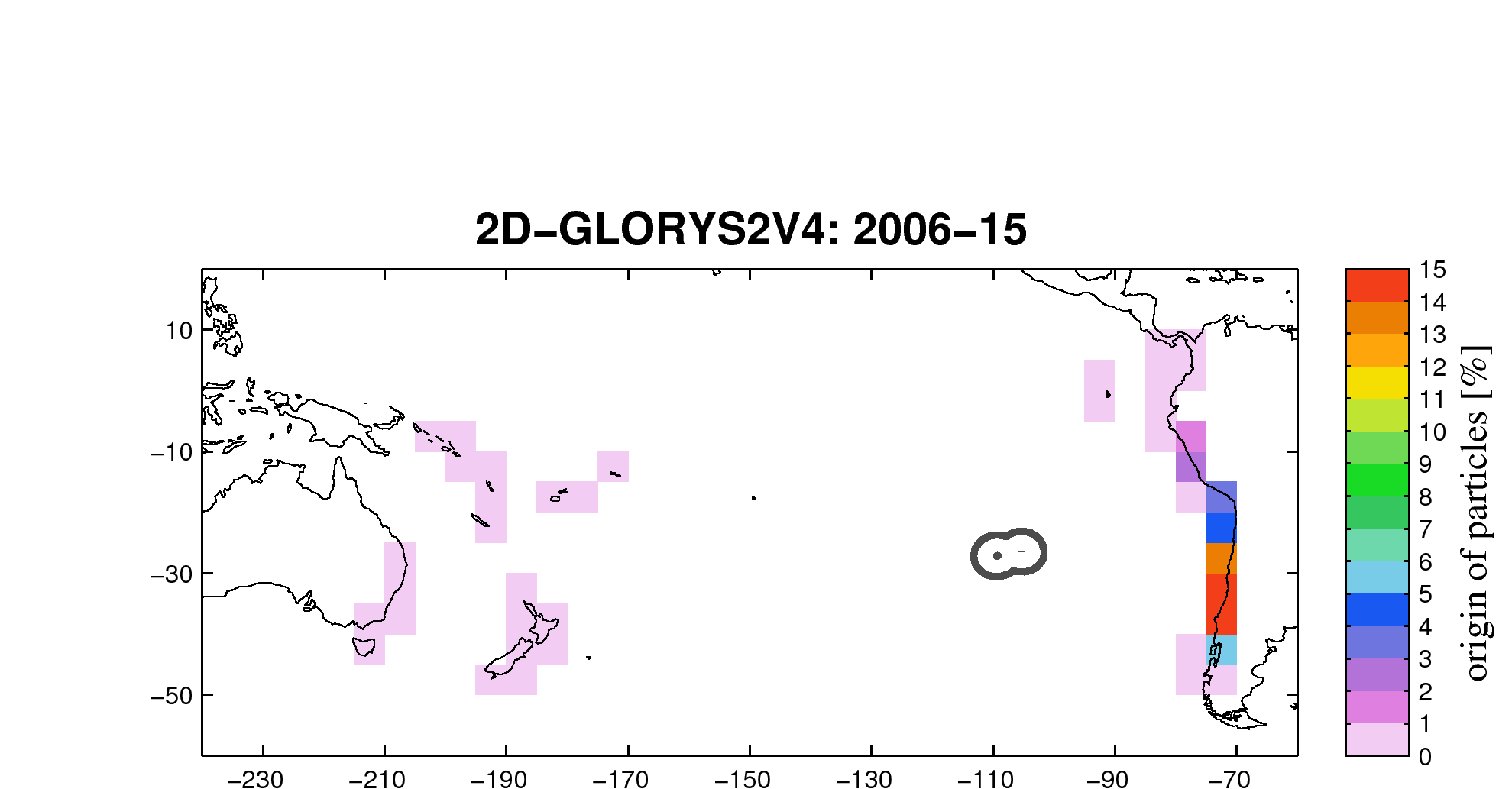


Figure S8: Connectivity strength (top) and time (bottom) of Easter Island with coastal regions of the Pacific basin obtained from experiments carried out in 2006-15 in the 2D flow for different model configurations: (left), Free NEMO 1/12 degree from NOC, (middle) Data Assimilated NEMO 1/4 degree (GLORYS2V4) from MERCATOR (right) Data Assimilated NEMO 1/12 degree from MERCATOR (GLORYS12). These analyses use the surface current of the mode


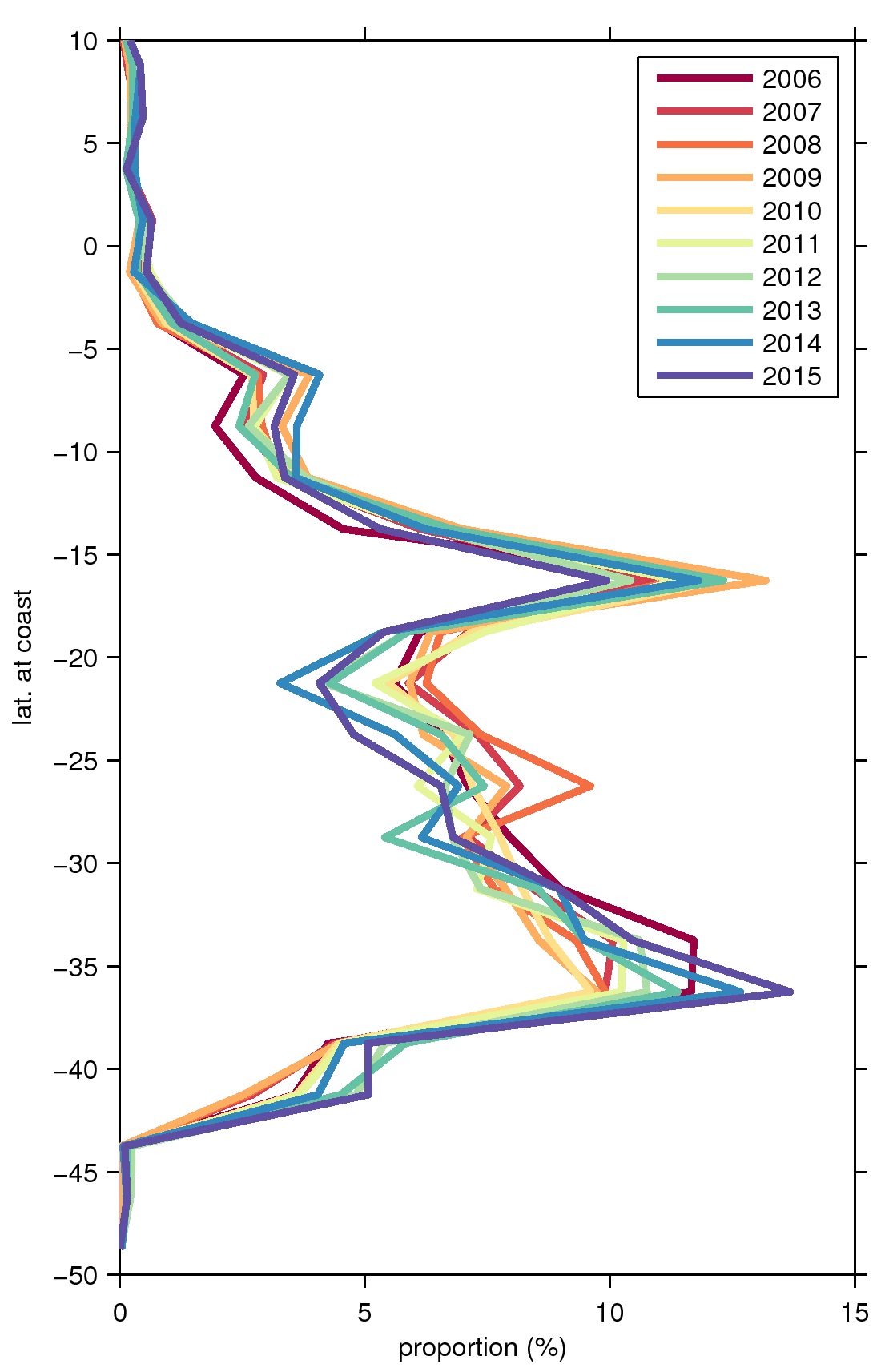


Figure S9: Percentage of particles, deployed within Easter Island Ecoregion and connecting to the South American coast for experiments carried out in different years within the decade 2006-2015. Note that within each year particles are released monthly and advected backwards for 5 years.
